# Methotrexate and cardiovascular prevention: an appraisal of the current evidence

**DOI:** 10.1177/17539447231215213

**Published:** 2023-12-20

**Authors:** Arduino A. Mangoni, Salvatore Sotgia, Angelo Zinellu, Ciriaco Carru, Gianfranco Pintus, Giovanni Damiani, Gian Luca Erre, Sara Tommasi

**Affiliations:** Discipline of Clinical Pharmacology, College of Medicine and Public Health, Flinders University, Bedford Park, SA 5042, Australia; Department of Clinical Pharmacology, Flinders Medical Centre, Southern Adelaide Local Health Network, Bedford Park, SA 5042, Australia; Department of Biomedical Sciences, University of Sassari, Sassari, Italy; Quality Control Unit, University Hospital (AOUSS), Sassari, Italy; Department of Biomedical Sciences, University of Sassari, Sassari, Italy; Quality Control Unit, University Hospital (AOUSS), Sassari, Italy; Department of Biomedical Sciences, University of Sassari, Sassari, Italy; Quality Control Unit, University Hospital (AOUSS), Sassari, Italy; Department of Biomedical Sciences, University of Sassari, Sassari, Italy; Quality Control Unit, University Hospital (AOUSS), Sassari, Italy; Department of Biomedical, Surgical and Dental Sciences, University of Milan, Milan, Italy; Italian Centre of Precision Medicine and Chronic Inflammation, Milan, Italy; Rheumatology Unit, Department of Clinical and Experimental Medicine, University Hospital (AOUSS) and University of Sassari, Sassari, Italy; Discipline of Clinical Pharmacology, College of Medicine and Public Health, Flinders University, Adelaide, SA, Australia; Department of Clinical Pharmacology, Flinders Medical Centre, Southern Adelaide Local Health Network, Adelaide, SA, Australia

**Keywords:** atherosclerosis, heart disease risk factors, immunity, inflammation, methotrexate

## Abstract

New evidence continues to accumulate regarding a significant association between excessive inflammation and dysregulated immunity (local and systemic) and the risk of cardiovascular events in different patient cohorts. Whilst research has sought to identify novel atheroprotective therapies targeting inflammation and immunity, several marketed drugs for rheumatological conditions may serve a similar purpose. One such drug, methotrexate, has been used since 1948 for treating cancer and, more recently, for a wide range of dysimmune conditions. Over the last 30 years, epidemiological and experimental studies have shown that methotrexate is independently associated with a reduced risk of cardiovascular disease, particularly in rheumatological patients, and exerts several beneficial effects on vascular homeostasis and blood pressure control. This review article discusses the current challenges with managing cardiovascular risk and the new frontiers offered by drug discovery and drug repurposing targeting inflammation and immunity with a focus on methotrexate. Specifically, the article critically appraises the results of observational, cross-sectional and intervention studies investigating the effects of methotrexate on overall cardiovascular risk and individual risk factors. It also discusses the putative molecular mechanisms underpinning the atheroprotective effects of methotrexate and the practical advantages of using methotrexate in cardiovascular prevention, and highlights future research directions in this area.

## Introduction

Atherosclerosis is a progressive disease that is characterized by the abnormal accumulation of lipids and fibrous elements in the arterial wall, with a consequent narrowing of the arterial lumen.^1^ The primary clinical manifestations of atherosclerosis, ischaemic heart disease and stroke, remain the leading cause of disability, mortality and excessive healthcare costs worldwide.^2^ For example, in the Global Burden of Disease 2019 study, a multinational research collaboration, the global prevalence of cardiovascular disease increased from 271 million in 1990 to 523 million in 2019.^3^ During the same period, there was also a significant increase in cardiovascular deaths, from 12.1 to 18.6 million, and a doubling of years lived with disability, from 17.7 to 34.4 million, due to ischaemic heart disease and stroke.^3^ Therefore, despite the availability of medications targeting key neurohormonal pathways (e.g. the renin–angiotensin–aldosterone system), blood pressure, lipid profile and platelet aggregation, additional research is needed to investigate the significance of unconventional cellular and biochemical mechanisms underpinning atherosclerosis. There is robust evidence that such mechanisms involve or even trigger the dysregulation of the immune system and the excessive activation of specific inflammatory pathways.^4–7^

A chronic local (arterial wall) and systemic pro-inflammatory and pro-oxidant state associated with a dysregulation of immune pathways plays a critical role in the pathophysiology of atherosclerosis.^8^ Such alterations also mediate the untoward effects of established cardiovascular risk factors, particularly diabetes,^9–11^ arterial hypertension,^12,13^ hypercholesterolaemia^14–18^ and the metabolic syndrome.^19,20^ Consequently, there has been an increasing focus on discovering novel immunomodulatory and anti-inflammatory agents with atheroprotective effects that could complement existing cardiovascular therapies.^21–23^ An alternative approach consists of determining the atheroprotective potential of other traditional immunomodulatory and anti-inflammatory agents that are commonly prescribed in patients with autoimmune and/or inflammatory conditions.^24–26^ If effective, such ‘drug repurposing’ approaches would minimize the costs and uncertainties of conventional drug discovery programmes and provide rapid public health benefits in cardiovascular prevention, defined as the combination of pharmacological and non-pharmacological strategies used to reduce the risk of cardiovascular disease in the population.^27,28^

One example of such a traditional immunomodulatory and anti-inflammatory drug is methotrexate, a pteridine analogue that was initially used as an anti-cancer agent and, more recently, as a conventional synthetic disease-modifying anti-rheumatic drug (csDMARD).^29–36^ Evidence generated from experimental and observational clinical studies conducted over the last 30 years suggests that treatment with methotrexate is also associated with beneficial effects on surrogate markers of atherosclerosis and cardiovascular clinical endpoints, for example, myocardial infarction and stroke.^37,38^ Therefore, methotrexate could be a suitable candidate for ‘drug repurposing’ strategies aimed at enhancing the efficacy of national and international cardiovascular prevention programmes.

This review article discusses the issues that limit the efficacy of existing cardiovascular prevention strategies, particularly residual cardiovascular and inflammatory risk, and the critical pathophysiological role of dysregulated immunity and inflammation in driving the onset and the progression of atherosclerosis. Then, it critically appraises the published evidence, focusing on studies conducted over the last 5 years, regarding the potential atheroprotective effects of methotrexate in experimental and clinical studies in patients with and without autoimmune and inflammatory conditions. Finally, it discusses the potential practical advantages of methotrexate therapy over available treatments for routine cardiovascular prevention and proposes new research directions in this area, including the design of future intervention studies investigating the effects of methotrexate on cardiovascular risk.

## Residual cardiovascular and inflammatory risk

A significant number of patients with previous atherosclerotic cardiovascular events, for example, acute coronary syndrome and ischaemic stroke, suffer from further events despite maximal treatment with statins, beta-blockers, antiplatelet agents, anticoagulants, angiotensin-converting enzyme (ACE) inhibitors and angiotensin receptor blockers.^39,40^ This observation suggests the presence of a significant ‘residual cardiovascular risk’, that is, the component of an individual patient’s cardiovascular risk that is not influenced by existing treatments.^41–43^ The increasing recognition of the critical role played by excess inflammation and dysregulated immunity in driving this residual risk has led several experts to rename this phenomenon ‘residual inflammatory risk’. The results of several observational studies support the clinical relevance of the residual inflammatory risk, potentially ascribable to the local pool of pro-inflammatory T memory cells.^44–46^ For example, in a Chinese study of 5840 patients with a recent ischaemic stroke or transient ischaemic attack receiving optimal preventive treatment, the subgroup with relatively high C-reactive protein (CRP) concentrations both at baseline and at 3-month of follow-up had significantly worse outcomes at 1 year when compared to the subgroup with relatively low CRP at both timepoints (stroke recurrence: adjusted hazard ratio, aHR = 1.39, 95% CI: 1.08–1.78, *p* = 0.01; composite of stroke, myocardial infarction, and cardiovascular death: aHR = 1.43, 95% CI: 1.12–1.82, *p* = 0.004; all-cause mortality: aHR = 2.57, 95% CI: 1.50–4.41, *p* < 0.001; and poor functional outcome: aHR = 1.75, 95% CI: 1.34–2.28, *p* < 0.001).^47^ In another study of 3013 patients undergoing percutaneous coronary revascularisation and receiving optimal preventive treatment, those with persistently high CRP at baseline and after 4 weeks had a significantly higher risk of major adverse cardiac and cerebrovascular events at 1 year when compared with those with low CRP at both timepoints (aHR = 2.10, 95% CI: 1.45–3.02, *p* < 0.001). Different risks were also observed in two other subgroups (low baseline and high CRP at follow-up: aHR = 1.91, 95% CI: 1.21–3.03, *p* = 0.006; high baseline and low CRP at follow-up: aHR = 1.52, 95% CI: 0.95–2.44, *p* = 0.08), suggesting the importance of the temporal direction in changes in inflammation in modulating residual inflammatory risk.^48^ Several other recent observational trials and post-hoc analyses of intervention trials have similarly reported significant associations between residual inflammatory risk, assessed by measuring CRP or other biomarkers (e.g. the neutrophil-to-lymphocyte ratio), and adverse outcomes in patients with atherosclerosis.^49–55^

## Atheroprotective strategies targeting inflammation and immunity

The structural and functional integrity of the endothelium is critical to ensure the maintenance of homeostatic mechanisms protecting against atherosclerosis, mainly through the production of the endogenous messenger nitric oxide (NO) by endothelial NO synthase (eNOS). NO regulates endothelial-dependent vasodilation, peripheral vascular resistance, arterial stiffness, blood pressure and platelet activity, preventing at the same time the adhesion of leucocytes to the arterial wall and the proliferation of vascular smooth muscle cells, critical steps involved in the pathophysiology of atherosclerosis.^56–63^ According to the ‘inflammatory theory of atherosclerosis’ postulated some 40 years ago, the dysregulated production of specific cytokines by subpopulations of macrophages (M1), for example, tumour necrosis factor-alpha (TNF-α), interleukin-6 (IL-6) and IL-12, favours excess inflammation in the arterial wall. This, in turn, favours the oxidation of specific cholesterol fractions, that is, low-density lipoprotein (LDL) cholesterol, endothelial damage, the creation of foam cells and the ultimate formation of the atherosclerotic plaque.^17,21,22,64^

In support of the pathophysiological role played by pro-inflammatory cytokines in atherosclerosis, several observational studies have reported significant associations between circulating cytokines and the risk of adverse cardiovascular outcomes. For example, in a study of patients with myocardial infarction, the concentrations of TNF-α measured after an average of 9 months after the event were significantly higher in those who experienced recurrent cardiovascular events during follow-up than those who did not (2.84 pg/mL *versus* 2.57 pg/mL, *p* = 0.02). Notably, the association between TNF-α concentrations and recurrent events was also independent of conventional cardiovascular risk factors.^65^ Similarly, a recent two-sample Mendelian randomization study has reported significant associations between genetically predicted TNF-α concentrations and ischaemic heart disease (odds ratio, OR = 2.25, 95% CI: 1.50–3.37, *p* < 0.001) and stroke (OR: 2.27, 95% CI: 1.50–3.43, *p* < 0.001).^66^ In another prospective study investigating 3269 patients with acute coronary syndrome, plasma IL-6 concentrations ⩾5 ng/L were significantly associated with 12-month mortality in patients not undergoing revascularisation (relative risk, RR = 3.47, 95% CI: 1.95–6.21, *p* < 0.001) but not in those undergoing revascularisation (RR = 1.43, 95% CI: 0.64–3.21, *p* = 0.38).^67^ Taken together, these studies highlight that the negative impact of excess inflammation is not only limited to the assessment of surrogate markers in experimental studies but also translates into a tangible increase in cardiovascular risk at the population level.

This evidence has stimulated a significant body of research over the last 10–15 years that has led to the identification of promising atheroprotective treatments targeting specific immune and inflammatory mediators. Such mediators include interleukin-1β (e.g. canakinumab),^65^ the interleukin-1 receptor (e.g. anakinra),^68^ the NLR family pyrin domain containing three inflammasome (e.g. MCC950 and tranilast),^69,70^ TNF-α (e.g. adalimumab),^71^ IL-6 (e.g. tocilizumab),^72^ chemokines (e.g. maraviroc and MNL1202),^73,74^ interleukin-2 (e.g. aldesleukin)^75^ and CD20 (e.g. rituximab).^76^ However, it is essential to highlight that these and other agents under investigation are often characterized by prohibitive costs and toxicity,^77–83^ which may limit their widespread use in cardiovascular prevention, a type of treatment that can last for several decades. Recent randomized-controlled studies have also demonstrated the benefits of ‘drug repurposing’ strategies for combating atherosclerosis with colchicine, a relatively old antimalarial, antimitotic and anti-inflammatory agent targeting multiple cellular pathways that is used for acute gout and pericarditis.^84,85^ A similar drug repurposing approach in the quest for alternative atheroprotective treatments will be discussed for another traditional immunomodulatory and anti-inflammatory agent, methotrexate, in the following sections.

## Methotrexate pharmacology and role in cardiovascular risk in clinical studies

Methotrexate, a pteridine molecule and an analogue of the B-vitamin folic acid, has been used since 1948 for the treatment of cancer and, more recently over the last 30–40 years, as a csDMARD for a wide range of autoimmune and autoinflammatory conditions.^34,36,86–88^ In this context, it is essential to emphasize that the doses of methotrexate used for the treatment of autoimmune and inflammatory conditions, between 7.5 and 30 mg weekly, are considerably lower than those used for malignancies, up to ⩾500 mg/m^2^.^34,86–90^

### Pharmacology of methotrexate

**Figure 1.**
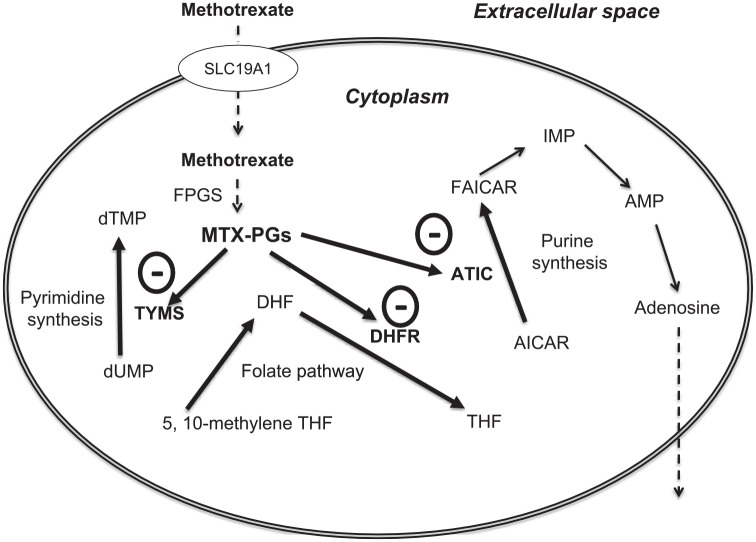
The pharmacology of methotrexate. AICAR, 5-aminoimidazole-4-carboxamide ribonucleoside; AMP, adenosine monophosphate; ATIC, aminoimidazole carboxamide ribonucleotide transformylase/inosine monophosphate cyclohydrolase; DHF, dihydrofolate; DHFR, dihydrofolate reductase; dTMP, deoxythymidine monophosphate; dUMP, deoxyuridine monophosphate; FAICAR, 5-formamidoimidazole-4-carboxamide ribotide; FPGS, folylpolyglutamate synthetase; IMP, inosine monophosphate; MTX-PGs, methotrexate polyglutamates; SLC19A1, solute carrier family 19 member 1; THF, tetrahydrofolate; TYMS, thymidylate synthase.

In patients with autoimmune and/or inflammatory conditions, methotrexate is normally administered once weekly either orally or subcutaneously. The mean bioavailability following oral administration has been shown to be 0.64 (range 0.21–0.96) when compared to subcutaneous administration.^91^ Subcutaneous methotrexate is gaining increasing popularity in clinical practice because of the higher bioavailability, as previously described, a more predictable pharmacokinetic profile, and a reduced rate of gastrointestinal toxicity when compared to oral methotrexate.^92^ Circulating methotrexate is not significantly bound to plasma proteins, ~50%, can easily distribute in the synovial fluid and is primarily eliminated by the kidney through glomerular filtration and active tubular secretion.^93,94^ The plasma half-life of methotrexate ranges between 4.5 and 10 h.^93,94^ However, the circulating concentrations of methotrexate are not particularly significant from a clinical standpoint as the drug enters cells *via* the human solute carrier superfamily of transporters before accumulating as pharmacologically active polyglutamate forms by folylpolyglutamate synthetases (Figure 1).^95^ A pharmacokinetic study has shown that the median time to achieve a steady state of the different forms of methotrexate polyglutamate concentrations in red blood cells after commencing oral methotrexate ranged between 6 and 149 weeks.^96^ The same study reported that the median time for the polyglutamates to become undetectable following treatment cessation ranged between 4 and 10 weeks.^96^ This period is considerably longer that the half-life of circulating methotrexate, which also justifies the weekly administration schedules in patients with autoimmune and inflammatory disorders.

**Figure 2.**
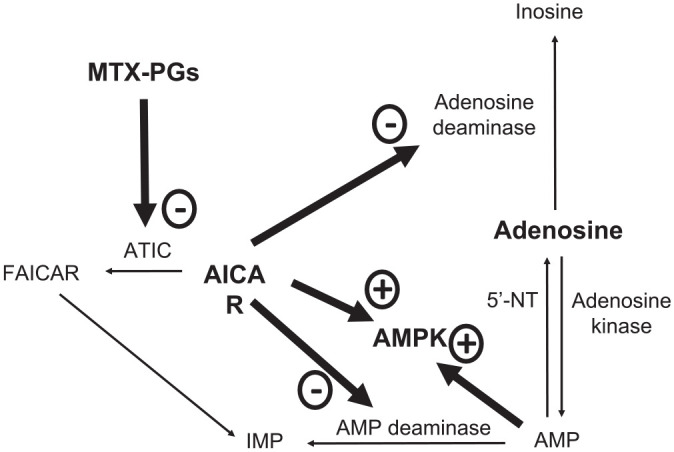
The effects of methotrexate on adenosine, AICAR and AMPK. AICAR, 5-aminoimidazole-4-carboxamide ribonucleoside; AMP, adenosine monophosphate; AMPK, 5′ adenosine monophosphate-activated protein kinase; ATIC, aminoimidazole carboxamide ribonucleotide transformylase/inosine monophosphate cyclohydrolase; FAICAR, 5-formamidoimidazole-4-carboxamide ribotide; IMP, inosine monophosphate; MTX-PGs, methotrexate polyglutamates; 5′-NT, 5′-Nucleotidase.

The polyglutamate forms mediate the inhibitory effects of methotrexate on the biosynthesis of purines and pyrimidines. These effects involve the inhibition of the enzymes thymidylate synthase, dihydrofolate reductase and aminoimidazole carboxamide ribonucleotide (AICAR) transformylase (Figure 1) (ATIC).^97^ The accumulation of the ATIC substrate, AICAR, in turn, favours the accumulation of adenosine, a critical anti-inflammatory mediator, through the inhibition of catabolic pathways mediated by adenosine deaminase and adenosine monophosphate deaminase (Figures 1 and 2).^97^

Treatment with methotrexate, particularly long-term, is known to be associated with gastroenterological, haematological, renal, neurological, pulmonary and mucocutaneous toxicity of different severity.^98,99^ However, with appropriate dosing and regular clinical and biochemical monitoring, the clinical manifestations of toxicity appear to be relatively infrequent and overall benign. For example, in an observational study of 673 patients with inflammatory arthritis, including rheumatoid arthritis, about three-quarters remained on methotrexate after 5 years. In the subgroup that stopped treatment, 11% reported inefficacy and opted for withdrawal, 6% liver abnormalities and a further 6% haematological abnormalities.^100^ In a study of 379 patients with rheumatoid arthritis receiving 1-year treatment with methotrexate with other csDMARDs and/or corticosteroids, only 2% reported serious adverse events.^101^ In a randomized-controlled study investigating the effects of 1-year treatment with methotrexate *versus* placebo in patients with arthritis thought to progress to rheumatoid arthritis, there were non-significant between-group differences in serious adverse events (11% *versus* 11%). However, patients receiving methotrexate had a higher incidence of significant (>3 × upper limit of normal) elevations in liver enzymes (incidence rate per 100 persons-years: 6.1, 95% CI: 3.2–10.4 *versus* 0.5, 95% CI: 0.0–27.4, *p* = 0.0025).^102^ Additionally, early reports suggesting an increased risk of liver fibrosis during methotrexate treatment have not been confirmed in recent studies.^103,104^ The safety profile of methotrexate was also comprehensively investigated in a secondary analysis of the Cardiovascular Inflammation Reduction Trial (CIRT). The authors reported a mildly yet significantly higher 3-year cumulative incidence of severe adverse events in patients receiving methotrexate *versus* placebo (0.13, 95% CI: 0.12–0.15 *versus* 0.10, 95% CI: 0.08–0.11).^105^ The safety of methotrexate reported in these studies appears similar to that reported in trials of conventional atheroprotective drugs. For example, in the Systolic Blood Pressure Intervention Trial in 9069 patients, 9.8% in the intensive treatment arm and 7.2% in the standard treatment arm reported serious adverse events.^106^ Furthermore, in the Ongoing Telmisartan Alone and in Combination with Ramipril Global Endpoint Trial investigating a total of 25,620 patients, the incidence of serious adverse events in the three treatment arms ranged between 5.0% and 12.0%.^107^

### Rheumatoid arthritis and cardiovascular disease

The pathophysiological mechanisms underlying one of the most common and studied types of autoimmune disease, rheumatoid arthritis,^108^ exhibit significant similarities with atherosclerosis. Such similarities include the presence of a vascular and systemic pro-inflammatory and pro-oxidant state,^109–115^ reduced NO synthesis and endothelial dysfunction,^116–120^ arterial stiffening^121–124^ and an increased risk of arterial hypertension.^125,126^ A critical additional element of similarity is represented by the excess production of pro-inflammatory and pro-atherogenic cytokines, for example, TNF-α, IL-1 and IL-6, that synergistically build and maintain a pro-inflammatory microenvironment in blood vessels.^127–130^ Not surprisingly, the risk of atherosclerotic cardiovascular disease in this group is significantly higher than the general population, as also shown in a systematic review and meta-analysis of six studies (RR = 1.55, 95% CI: 1.18–2.02). In subgroup analysis, the RR was higher in patients aged <60 years (RR = 1.98, 95% CI: 1.41–2.79) than in those aged ⩾60 years (RR = 1.43, 95% CI: 1.16–1.75), although the risk remained significant in both groups.^131^ In addition to inflammation and oxidative stress, traditional risk factors, for example, diabetes, arterial hypertension and the metabolic syndrome, have also been shown to account for the increased risk of atherosclerosis and cardiovascular disease in rheumatoid arthritis.^132,133^

### Methotrexate and cardiovascular risk

Several observational studies have reported that treatment with methotrexate is associated with a significant reduction of cardiovascular and all-cause mortality in patients with rheumatoid arthritis. For example, in a recent systematic review and meta-analysis of 15 studies, 7 longitudinal observational cohort, 6 retrospective cohort and 2 prospective cohort studies, the use of methotrexate was associated with a significant reduction in all-cause mortality (HR = 0.59, 95% CI: 0.50–0.71, *p* < 0.001). In a subgroup analysis of four studies, the use of methotrexate was also associated with a significant reduction in cardiovascular mortality (HR = 0.72, 95% CI: 0.53–0.97, *p* = 0.031).^134^ The results of another systematic review and meta-analysis also support the possible protective role of methotrexate against cardiovascular events in rheumatoid arthritis. In 10 selected studies, 3 cohort, 2 cross-sectional, 1 case–control and four nest case–control, investigating a total of 195,416 participants, there was a significant negative association between methotrexate and cardiovascular events (RR = 0.80, 95% CI: 0.73–0.88, *p* < 0.001). The association was similar in a subgroup of eight studies that adjusted for concomitant cardiovascular risk factors (RR = 0.78, 95% CI: 0.71–0.86, *p* = 0.003).^135^

Importantly, current evidence suggests that other immunomodulatory and anti-inflammatory agents do not share the putative protective effects of methotrexate against atherosclerosis and cardiovascular disease.^136,137^ In a systematic review and meta-analysis of eight studies investigating a total of 65,736 patients with rheumatoid arthritis, methotrexate use was associated with a significant reduction in total cardiovascular events when compared to other csDMARDs (RR = 0.72, 95% CI: 0.57–0.91, *p* = 0.007). A substantial reduction in the specific risk of myocardial infarction was also observed in a subgroup of three studies (RR = 0.81, 95% CI: 0.68–0.96).^138^ A more recent retrospective cohort study assessing Medicare claims data in the United States for the period 2006–2015 sought to investigate whether methotrexate use might exert atheroprotective effects in patients with rheumatoid arthritis who are already receiving treatment with biologic agents. In a total of 88,255 patients receiving biologics, the additional use of methotrexate was associated with a significant reduction in the risk of a composite endpoint of myocardial infarction, stroke and fatal cardiovascular disease (aHR = 0.76, 95% CI: 0.68–0.85).^139^ The effect size of the cardiovascular risk reduction with methotrexate in these studies is comparable to that observed in intervention studies of established atheroprotective agents, for example, ACE inhibitors (RR = 0.80, 95% CI: 0.70–0.91),^140^ and statins (OR = 0.69, 95% CI: 0.64–0.75).^141^

Another recent study has sought to investigate whether methotrexate may have competitive advantages in terms of cardiovascular prevention over other csDMARDs, such as hydroxychloroquine, an agent with evidence of atheroprotective effects in animal and human studies.^26,142^ Using Medicare data during the period 2008–2016, the study authors propensity score-matched 54,462 patients with rheumatoid arthritis aged ⩾65 years commenced on either hydroxychloroquine or methotrexate. No significant between-group differences were observed in the primary endpoint, sudden cardiac arrest (or ventricular arrhythmia) and major adverse cardiovascular events. However, in a subgroup of patients with heart failure, hydroxychloroquine was associated with a significantly higher risk of major adverse cardiovascular events (HR = 1.30, 95% CI: 1.08–1.56), cardiovascular mortality (HR = 1.34, 95% CI: 1.06–1.70), all-cause mortality (HR = 1.22, 95% CI: 1.04–1.43), myocardial infarction (HR = 1.74, 95% CI: 1.25–2.42) and hospitalizations for heart failure (HR = 1.29, 95% CI: 1.07–1.54) when compared to methotrexate.^143^

The main study assessing the effects of methotrexate on cardiovascular prevention, the CIRT, randomized 4786 patients without autoimmune conditions but with myocardial infarction or multivessel coronary disease with either type 2 diabetes or metabolic syndrome to methotrexate (target dose of 15–20 mg/week) or placebo. There were non-significant between-group differences in the primary endpoint, a composite of nonfatal myocardial infarction, nonfatal stroke, cardiovascular death and hospitalization for unstable angina requiring urgent revascularisation (HR = 0.96, 95% CI: 0.79–1.16).^144^ Whilst the results of this study do not support the presence of significant atheroprotective effects of methotrexate in patients without autoimmune conditions, it is essential to emphasize that, by trial design, both patients in the methotrexate and the placebo arms received treatment with the B-vitamin folic acid. Folic acid is often co-administered with methotrexate by rheumatologists, given that both compounds compete for the same transporter in the intestine and cellular uptake and that folic acid supplementation has been shown to reduce the incidence of adverse effects with methotrexate.^145–147^ However, at the same time, there is robust evidence from experimental and clinical studies that treatment with folic acid *per se* exerts significant atheroprotective effects, including the lowering of the highly reactive and pro-atherogenic amino acid homocysteine,^148^ improved NO synthesis and endothelial function^149^ and reduced arterial stiffness and blood pressure.^150,151^ Furthermore, in a large randomized-controlled trial conducted in China in 20,702 hypertensive patients without previous myocardial infarction or stroke, a combination treatment of folic acid with the ACE inhibitor enalapril significantly reduced the risk of overall stroke (primary endpoint, HR = 0.79, 95% CI: 0.68–0.93); first ischaemic stroke (HR = 0.76, 95% CI: 0.64–0.91) and a composite endpoint of cardiovascular death, myocardial infarction and stroke (HR = 0.80, 95% CI: 0.69–0.92). By contrast, there were non-significant between-group differences in other secondary endpoints, that is, haemorrhagic stroke (HR = 0.93, 95% CI: 0.65–1.34), myocardial infarction (HR = 1.04, 95% CI: 0.60–1.82) and all-cause mortality (HR = 0.94, 95% CI: 0.81–1.10).^152^ Therefore, further studies investigating the effects of methotrexate on cardiovascular prevention are warranted to determine whether the use of folic acid in the comparator group might have diluted the potential atheroprotective effects of methotrexate in the CIRT study.

## Effects of methotrexate on traditional cardiovascular risk factors

**Figure 3.**
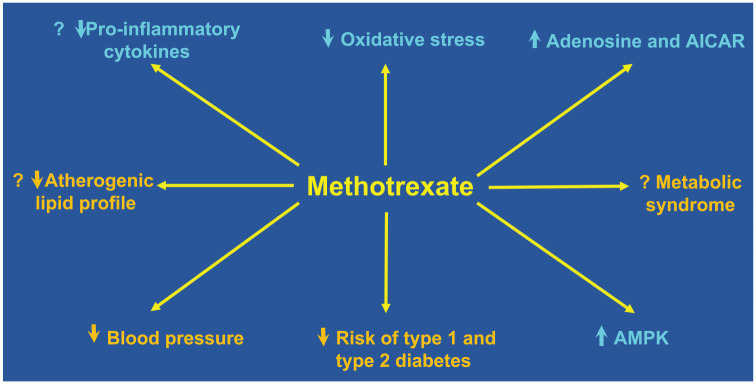
Effects of methotrexate on cardiovascular risk factors (orange) and putative atheroprotective mechanisms (cyan) according to experimental and clinical studies. AICAR, 5-aminoimidazole-4-carboxamide ribonucleoside; AMPK, 5′ adenosine monophosphate-activated protein kinase.

Several experimental and clinical studies have investigated the effects of methotrexate on conventional cardiovascular risk factors, particularly dyslipidaemia, diabetes, arterial hypertension and the metabolic syndrome (Figure 3).

### Lipid profile

In a study in cholesterol-fed rabbits, treatment with lipid core nanoparticles containing methotrexate, with or without paclitaxel, caused significant regression of aortic plaque (−59%) and intima areas (−57%). These effects were associated with a substantial reduction in macrophages and TNF-α gene expression.^153^ In a secondary analysis of the Optimised Treatment Algorithm for Patients With Early Rheumatoid Arthritis (OPERA) trial, comparing the effects of adalimumab and methotrexate (*n* = 86) *versus* methotrexate and placebo (*n* = 88), there was no significant between-group difference in the changes at 1 year *versus* baseline in total, LDL cholesterol, high-density lipoprotein (HDL) cholesterol and triglyceride concentrations.^154^ In an observational study of 262 patients with psoriasis, 12-week treatment with methotrexate significantly reduced total cholesterol (*p* < 0.05), LDL cholesterol (*p* < 0.05), apolipoprotein B (*p* < 0.001) and lipoprotein A (*p* < 0.001) in annexin A6, a protein that regulates cholesterol homeostasis,^155^ TC and CC genotype carriers of *rs11960458* and apolipoprotein B (*p* = 0.04) in TT genotype carriers. However, methotrexate also significantly reduced the concentrations of the atheroprotective subfractions, HDL cholesterol (*p* = 0.007) and apolipoprotein A1 (*p* = 0.04) in TC genotype carriers of *rs11960458*. Moreover, methotrexate significantly reduced triglyceride concentrations only in the CC genotype carriers (*p* = 0.01).^156^ In another study of 288 patients with psoriasis, 136 with and 152 without psoriatic arthritis, 12-week treatment with methotrexate significantly lowered apolipoprotein B (*p* = 0.0003), total cholesterol (*p* = 0.0007), triglycerides (*p* = 0.04), HDL cholesterol (*p* = 0.037) and lipoprotein A (*p* = 0.005) in patients with arthritis, and apolipoprotein B (*p* < 0.0001), total cholesterol (*p* < 0.0001), HDL cholesterol (*p* = 0.011), LDL cholesterol (*p* = 0.0001) and lipoprotein A (*p* < 0.0001) in patients without arthritis.^157^ Interestingly, in another study in 35 patients with psoriasis, 12-week treatment with methotrexate significantly reduced the concentrations of proprotein convertase subtilisin/kexin type 9,^158^ critically involved in cholesterol homeostasis by binding to the LDL receptor in hepatocytes and an established therapeutic target for cardiovascular prevention.^159,160^ Collectively, these studies have provided conflicting results on the effects of methotrexate treatment on lipid profile and, expressly, on atheroprotective *versus* pro-atherogenic cholesterol fractions (Figure 3).

### Type 1 and type 2 diabetes

In a systematic review and meta-analysis of 16 studies investigating patients with rheumatoid arthritis, 3 with a cross-sectional design, 1 with a nested case-control design, 8 prospective cohorts and 4 retrospective cohorts, the use of methotrexate was associated with a significant reduction in the risk of type 2 diabetes (RR = 0.13, 95% CI: 0.08–0.22). Factors significantly associated with the reduced risk of diabetes included age >60 years, rheumatoid arthritis duration ⩽2 years and the measurement of disease activity.^161^ A similar negative association between methotrexate and type 1 and type 2 diabetes has been reported in another systematic review and meta-analysis of 15 studies (seven on methotrexate) investigating a total of 552,019 patients with rheumatoid arthritis (HR = 0.81, 95% CI: 0.75–0.87). The reduced risk of type 1 and type 2 diabetes with methotrexate was similar in studies assessing comparisons with non-users of methotrexate (HR = 0.77, 95% CI: 0.67–0.88) as well as users of csDMARDs not including methotrexate or hydroxychloroquine (HR = 0.85, 95% CI: 0.74–0.98).^162^ Furthermore, in a nationwide population study of 69,799 patients with rheumatoid arthritis but without type 1 and type 2 diabetes at baseline, the long-term use of methotrexate (>270 days/year) was associated with a significant reduction in incident type 1 and type 2 diabetes (adjusted OR = 0.84, 95% CI: 0.78–0.92).^163^ Taken together, the available evidence suggests that methotrexate treatment in rheumatoid arthritis is associated with a reduced risk of type 1 and type 2 diabetes, although the mechanisms underpinning the effects on glucose metabolism require further studies (Figure 3).

### Arterial hypertension

In a repeated cross-sectional study of patients with rheumatoid arthritis, the use of methotrexate was associated with a significantly lower clinical and 24-h blood pressure when compared to other csDMARDs.^120^ Another study of 21,916 patients with rheumatoid arthritis with data from administrative Veterans Affairs databases in United States investigated the changes in blood pressure after commencing methotrexate, leflunomide, sulfasalazine, hydroxychloroquine, TNF-α inhibitors or prednisone. In this study, there was a reduction in blood pressure after starting prednisone, methotrexate and hydroxychloroquine and a more modest decline with sulfasalazine and tumour necrosis factor inhibitors. Notably, in patients commencing methotrexate, a more significant proportion had an optimal blood pressure control at 6 months *versus* baseline (51.0% *versus* 46.8%, *p* < 0.001). Similar associations were observed for prednisone, tumour necrosis factor inhibitors and hydroxychloroquine.^164^ Collectively, these studies suggest that methotrexate treatment can exert ameliorative effects on blood pressure control, at least in patients with rheumatoid arthritis (Figure 3).

### Metabolic syndrome

The association between methotrexate and the metabolic syndrome has been investigated in cross-sectional and intervention studies. In a cross-sectional study of 400 patients with rheumatoid arthritis, the use of methotrexate, but not other csDMARDs, was significantly and negatively associated with the risk of metabolic syndrome in multivariate regression analysis (adjusted OR = 0.52, 95% CI: 0.33–0.80, *p* = 0.004).^165^ The reduced prevalence of metabolic syndrome in methotrexate users *versus* non-users has also been reported in another cross-sectional study investigating 100 women with rheumatoid arthritis (17% *versus* 35%, *p* = 0.046).^166^ However, a retrospective study has failed to show any significant effect of 24-month methotrexate treatment on the prevalence of metabolic syndrome and its individual components in 70 patients with psoriatic arthritis.^167^ Therefore, there is conflicting evidence regarding the effects of methotrexate on the risk of metabolic syndrome and its individual components (Figure 3).

## Methotrexate and atheroprotection: Mechanistic insights

Several mechanisms have been postulated to account for the possible atheroprotective effects of methotrexate. Such mechanisms include cytokine modulation, the accumulation of adenosine, the activation of 5′ adenosine monophosphate-activated protein kinase (AMPK) and the regulation of the redox balance (Figure 3).

### Cytokines

Studies conducted over the last 20 years have shown the potential for methotrexate to downregulate several pro-inflammatory and pro-atherogenic cytokines, for example, TNF-α, IL-1 and IL-6,^168–175^ and to upregulate anti-atherogenic cytokines, for example, IL-10,^175–181^ providing a potential advantage over available therapies that target one pro-inflammatory cytokine. In more recent studies, the use of methotrexate-loaded chitosan nanoparticles has also been shown to significantly reduce the circulating concentrations of TNF-α (645 ± 37 pg/mL *versus* 140 ± 4 pg/mL, *p* < 0.001) and IL-6 (334 ± 34 pg/mL *versus* 62 ± 5 pg/mL, *p* < 0.001) in a rat model of arthritis.^182^ In another study, the release of another pro-inflammatory cytokine, IL-8, by monocytic MONO-MAC-6-cells activated by lysates of *Fusobacterium nucleatum* was significantly reduced by methotrexate, but not by other anti-inflammatory agents, that is, ibuprofen or prednisolone. However, the concentrations of IL-8 following treatment with methotrexate remained significantly higher than those measured in the absence of exposure to *F. nucleatum*.^183^ The results of another recent study have challenged the proposition that methotrexate can upregulate anti-inflammatory cytokines. In patients with plaque psoriasis, 12-week methotrexate treatment was associated with a significant reduction of the anti-inflammatory cytokine IL-10 (*p* = 0.02).^184^ Additional uncertainties regarding the clinical relevance of the modulation of cytokine pathways by methotrexate derive from a sub-analysis of the previously described CIRT study, which showed that methotrexate treatment failed to significantly reduce the concentrations of IL-1β and IL-6 during follow-up. Interestingly, in this study, methotrexate also failed to significantly reduce the concentrations of CRP. This observation might be related to the relatively low baseline concentrations of CRP (1.5 mg/L), IL-1β (1.5 pg/mL) and IL-6 (2.3 pg/mL) in CIRT participants, particularly when compared to other, successful, trials investigating treatments (canakinumab) targeting IL-1β and residual inflammatory risk (baseline CRP and IL-6 concentrations, 4.10 and 2.6 pg/mL, respectively).^144,185^

### Adenosine and AMPK

The methotrexate-mediated accumulation of adenosine (Figures 1 and 2) might exert significant effects on cardiovascular homeostasis, particularly vasodilatation through the inhibition of alpha-1 adrenergic vasoconstriction and the stimulation of the A_2A_ and A_2B_ receptors in the aorta,^186,187^ kidney^188^ and skeletal muscle.^189^ The resulting increase in blood flow in the renal medulla also favours natriuresis.^190^ The critical role of adenosine in maintaining cardiovascular homeostasis is further supported by studies reporting a significant increase in arterial stiffness and blood pressure following the pharmacological inhibition of the adenosine receptors, A_1_ and A_2A._^191,192^ There is also evidence that adenosine A_2B_ receptor activation prevents the formation of atherosclerotic lesions and reduces the plasma concentrations of cholesterol and triglycerides, possibly through the reduced activation of the transcription factor sterol regulatory element-binding protein 1 in the liver.^193,194^ Additional potential beneficial effects of adenosine include promoting the differentiation of monocytes into the anti-inflammatory M2 macrophage phenotype^195^ and upregulating cholesterol efflux transporters in macrophages. The transporters shown to be affected by adenosine include ABCA1, which effluxes cholesterol as apoA-1, a major component of HDL cholesterol, ABCG1, which effluxes cholesterol as HDL cholesterol, and sterol the cytochrome P450 enzyme 27-OH hydroxylase, which effluxes cholesterol in the form of 27-hydroxycholesterol.^196,197^ These forms of cholesterol prevent lipid overload and the transformation of macrophages in foam cells, which are critically involved in the formation and progression of the atherosclerotic plaque.^198^

AICAR *per se* can upregulate the AMPK,^199,200^ which protects endothelial cells against oxidative stress and apoptosis and inhibits vascular smooth muscle cell proliferation.^201–203^ There is also increasing evidence that AICAR and/or AMPK activation enhances vasodilation, reduces blood pressure and improves cholesterol efflux capacity.^204–211^ Furthermore, AMPK exerts beneficial effects on glucose homeostasis by stimulating cellular glucose uptake and glycolysis.^212–214^

### Redox balance

Methotrexate has been traditionally used at high doses to induce cytotoxicity in the treatment of malignancies as well as in experimental studies investigating the effects of rescuing treatments. The cytotoxic effects of methotrexate are ascribed to the inhibition of dihydrofolate reductase, involved in the conversion of dihydrofolate into tetrahydrofolate, required for the synthesis of the nucleotides of both DNA and RNA and the de novo purine synthesis of both purine and thymidylate synthase, which further inhibits DNA synthesis (Figure 1).^32,35,215^ However, at relatively high doses, methotrexate is also known to trigger a pro-oxidant state which further contributes to the structural and functional alteration of critical cell components, for example, lipids, proteins and DNA.^216^

Notably, however, studies have reported that at lower doses methotrexate can exert significant anti-oxidant effects. In one study, methotrexate treatment (2 μg) in HEK293 cells was able to directly scavenge free radicals, specifically O_2_^.−^, consequently inhibiting the formation of malondialdehyde–acetaldehyde adducts, including proteins exerting pro-inflammatory effects that have also been detected in atherosclerotic plaques.^217^ The use of a specific cell line to quantify the activation of the redox-sensitive transcription factor, nuclear factor (erythroid-derived 2)-like 2 (Nrf2), allowed to demonstrate that methotrexate also reduces the activation of the Nrf2-dependent intracellular redox signalling pathway.^218^ In a more recent study in rat primary astrocytes, pre-conditioning with 10 or 20 nM methotrexate (corresponding to 5 or 10 μg of the drug) enhanced the anti-oxidant defence mechanisms in these cells, expressed as the ratio of reduced to oxidized glutathione, as well as cell viability against a higher dose of methotrexate (500 nM or 0.23 mg/L).^219^ Taken together, the results of these studies suggest that, at specific doses, methotrexate can scavenge free radicals, reduce the activation of redox-sensitive intracellular signalling pathways and enhance anti-oxidant mechanisms in the context of atherosclerosis (Figure 3).

## Practical considerations for using methotrexate in cardiovascular prevention

One potential advantage of methotrexate, in terms of treatment adherence, is the once-weekly administration compared to the daily administration of currently available antihypertensives, antiplatelet and lipid-lowering agents. This feature is likely clinically relevant as poor treatment adherence remains a vexing issue in cardiovascular prevention. For example, a systematic review and meta-analysis of 45 prospective studies investigating a total of nearly 2 million patients reported a good adherence (defined as an intake of ⩾80%) to medications used for cardiovascular prevention in only 60% (95% CI: 52–68). In further analyses, patients with good adherence were significantly less likely to experience a cardiovascular event when compared to those with poor adherence (RR = 0.81, 95% CI: 0.76–0.86 for antihypertensive drugs).^220^

## Future research directions

In the last 5 years, additional evidence has accumulated on the association between the use of methotrexate, surrogate markers of atherosclerosis and hard cardiovascular endpoints in experimental and clinical studies (Figure 3). However, the contrasting nature of the results observed, particularly regarding the effects on cardiovascular morbidity and mortality in observational *versus* intervention studies, suggests that further research is warranted to support the repurposing of methotrexate for cardiovascular prevention (Table 1). In the first instance, studies should investigate the effects of this immunomodulatory and anti-inflammatory drug on a wide range of conventional cardiovascular risk factors and determine the mediating role of pro- and anti-inflammatory cytokines, adenosine, AMPK activation and oxidative stress. In this context, the negative results of the CIRT study, whilst disappointing, have been helpful for the design of future intervention trials for at least two reasons. Firstly, the relatively low-baseline CRP concentrations in study participants and the lack of tangible effects of methotrexate on the pro-inflammatory cytokines, IL-1β and IL-6, in CIRT suggests that further studies should focus on patients with significant residual inflammatory risk, that is, higher baseline CRP, IL-1β and IL-6 concentrations. Secondly, future studies should ideally investigate the effects of methotrexate separately from those of folic acid, given the previously described effects of this B-vitamin on endothelial function, blood pressure and other markers of atherosclerosis. Such trials should ideally investigate the impact of methotrexate alone, folic acid alone and methotrexate in combination with folic acid and include a non-methotrexate/folic acid comparator arm to fully determine the atheroprotective potential of methotrexate. Finally, the peculiar pharmacology of methotrexate, which involves membrane transporters for cellular uptake, enzymes for the biotransformation into intracellular polyglutamates and other enzymes as drug targets (Figure 1), has stimulated a significant body of research to investigate whether specific genetic characteristics can influence the efficacy and safety of the drug, particularly in rheumatological disorders.^221–223^ While pharmacogenetic studies investigating surrogate markers of atherosclerosis are in their infancy,^224^ the assessment of genetic polymorphisms might allow identifying specific subgroups that are more likely to benefit from the atheroprotective effects of methotrexate.

**Table 1.** Directions for future research to investigate the role of methotrexate in cardiovascular prevention.

• Study the effects of methotrexate on surrogate markers of atherosclerosis, risk, factors and cardiovascular endpoints independently of folic acid. • In intervention studies, include participants with high residual cardiovascular/inflammatory risk, defined using specific thresholds for CRP, IL-1 and IL-6 concentrations at baseline. • Investigate the mediating effects of pro-inflammatory and anti-inflammatory cytokines, adenosine, AICAR and redox balance. • Determine the potential role of genetic polymorphisms in specific transporters and enzymes in the identification of ‘high-responders’ *versus* ‘low-responders’ to the atheroprotective effects of methotrexate.

AICAR, 5-aminoimidazole-4-carboxamide ribonucleoside; CRP, C-reactive protein; IL, interleukin.

## Conclusion

The recognition that atherosclerosis is a chronic inflammatory disease of the arterial wall, and that significant residual cardiovascular/inflammatory risk exists in many patients despite maximal treatment with atheroprotective medications justifies the search for more effective therapies that target multiple pathways, including inflammation. Recent studies have highlighted the beneficial effects of new biologics targeting specific inflammatory pathways and traditional anti-inflammatory drugs with a broader range of effects, such as colchicine and hydroxychloroquine, in reducing cardiovascular events.^26,225–227^

This review has discussed the current evidence supporting the potential for methotrexate to be repurposed for the management of atherosclerotic cardiovascular disease in view of a unique combination of anti-inflammatory and, possibly, blood pressure lowering and vasculoprotective effects (Figure 3). However, one important limitation of this review is the lack of randomized-controlled studies demonstrating the efficacy of methotrexate in significantly reducing cardiovascular risk in patients with or without autoimmune and inflammatory disorders. In this context, the identification of patients that are most likely to benefit from methotrexate might require additional stratification by measuring pro-atherogenic and possibly anti-atherogenic, cytokine concentrations as well as genetic polymorphisms of relevant transporters and enzymes. Ultimately, however, only the completion of additional prospective studies that take into account the design considerations previously discussed will provide a definite answer regarding the therapeutic potential of methotrexate in cardiovascular prevention.

## Declarations

**Ethics approval and consent to participate:** Not applicable.

**Consent for publication:** Not applicable.

**Availability of data and materials:** Not applicable.

## References

[1] Lusis AJ . Atherosclerosis. Nature 2000; 407: 233–241. 11001066 10.1038/35025203 PMC2826222

[2] Herrington W Lacey B Sherliker P , et al. Epidemiology of atherosclerosis and the potential to reduce the global burden of atherothrombotic disease. Circ Res 2016; 118: 535–546. 26892956 10.1161/CIRCRESAHA.115.307611

[3] Roth GA Mensah GA Johnson CO . Global burden of cardiovascular diseases and risk factors, 1990. J Am Coll Cardiol 2020; 76: 2982–3021. 33309175 10.1016/j.jacc.2020.11.010 PMC7755038

[4] Weber C Habenicht AJR von Hundelshausen P . Novel mechanisms and therapeutic targets in atherosclerosis: inflammation and beyond. Eur Heart J 2023; 44: 2672–2681. 37210082 10.1093/eurheartj/ehad304

[5] Libby P Buring JE Badimon L , et al. Atherosclerosis. Nat Rev Dis Primers 2019; 5: 56. 31420554 10.1038/s41572-019-0106-z

[6] Frąk W Wojtasińska A Lisińska W , et al. Pathophysiology of cardiovascular diseases: new insights into molecular mechanisms of atherosclerosis, arterial hypertension, and coronary artery disease. Biomedicines 2022; 10: 1938. 36009488 10.3390/biomedicines10081938 PMC9405799

[7] Markin AM Sobenin IA Grechko AV , et al. Cellular mechanisms of human atherogenesis: focus on chronification of inflammation and mitochondrial mutations. Front Pharmacol 2020; 11: 642. 32528276 10.3389/fphar.2020.00642 PMC7247837

[8] Wolf D Ley K . Immunity and inflammation in atherosclerosis. Circ Res 2019; 124: 315–327. 30653442 10.1161/CIRCRESAHA.118.313591 PMC6342482

[9] Poznyak A Grechko AV Poggio P , et al. The diabetes mellitus-atherosclerosis connection: the role of lipid and glucose metabolism and chronic inflammation. Int J Mol Sci 2020; 21: 1835. 32155866 10.3390/ijms21051835 PMC7084712

[10] Edgar L Akbar N Braithwaite AT , et al. Hyperglycemia induces trained immunity in macrophages and their precursors and promotes atherosclerosis. Circulation 2021; 144: 961–982. 34255973 10.1161/CIRCULATIONAHA.120.046464 PMC8448412

[11] Clement CC Nanaware PP Yamazaki T , et al. Pleiotropic consequences of metabolic stress for the major histocompatibility complex class II molecule antigen processing and presentation machinery. Immunity 2021; 54: 721–736.e10. 10.1016/j.immuni.2021.02.019 PMC8046741 33725478

[12] Zhang Z Zhao L Zhou X , et al. Role of inflammation, immunity, and oxidative stress in hypertension: new insights and potential therapeutic targets. Front Immunol 2023; 13: 1098725. 36703963 10.3389/fimmu.2022.1098725 PMC9871625

[13] Madhur MS Elijovich F Alexander MR , et al. Hypertension: do inflammation and immunity hold the key to solving this epidemic? Circ Res 2021; 128: 908–933. 33793336 10.1161/CIRCRESAHA.121.318052 PMC8023750

[14] Galkina E Ley K . Immune and inflammatory mechanisms of atherosclerosis. Annu Rev Immunol 2009; 27: 165–197. 19302038 10.1146/annurev.immunol.021908.132620 PMC2734407

[15] Tall AR Yvan-Charvet L . Cholesterol, inflammation and innate immunity. Nat Rev Immunol 2015; 15: 104–116. 25614320 10.1038/nri3793 PMC4669071

[16] Aguilar-Ballester M Herrero-Cervera A Vinué Á , et al. Impact of cholesterol metabolism in immune cell function and atherosclerosis. Nutrients 2020; 12: 20200707. 10.3390/nu12072021 PMC7400846 32645995

[17] Libby P Ridker PM Maseri A . Inflammation and atherosclerosis. Circulation 2002; 105: 1135–1143. 11877368 10.1161/hc0902.104353

[18] Gimbrone MA Jr. García-Cardeña G . Endothelial cell dysfunction and the pathobiology of atherosclerosis. Circ Res 2016; 118: 620–636. 26892962 10.1161/CIRCRESAHA.115.306301 PMC4762052

[19] Monserrat-Mesquida M Quetglas-Llabrés M Capó X , et al. Metabolic syndrome is associated with oxidative stress and proinflammatory state. Antioxidants (Basel, Switzerland) 2020; 9: 236. 32178436 10.3390/antiox9030236 PMC7139344

[20] Manzoor MF Arif Z Kabir A , et al. Oxidative stress and metabolic diseases: Relevance and therapeutic strategies. Front Nutr 2022; 9: 994309. 36324618 10.3389/fnut.2022.994309 PMC9621294

[21] Kong P Cui ZY Huang XF , et al. Inflammation and atherosclerosis: signaling pathways and therapeutic intervention. Signal Transduct Target Ther 2022; 7: 131. 35459215 10.1038/s41392-022-00955-7 PMC9033871

[22] Soehnlein O Libby P . Targeting inflammation in atherosclerosis – from experimental insights to the clinic. Nat Rev Drug Discov 2021; 20: 589–610. 33976384 10.1038/s41573-021-00198-1 PMC8112476

[23] Engelen SE Robinson AJB Zurke YX , et al. Therapeutic strategies targeting inflammation and immunity in atherosclerosis: how to proceed? Nat Rev Cardiol 2022; 19: 522–542. 35102320 10.1038/s41569-021-00668-4 PMC8802279

[24] Chaffey L Roberti A Greaves DR . Drug repurposing in cardiovascular inflammation: successes, failures, and future opportunities. Front Pharmacol 2022; 13: 1046406. 36339576 10.3389/fphar.2022.1046406 PMC9634418

[25] Bouabdallaoui N Tardif JC . Repurposing colchicine for heart disease. Annu Rev Pharmacol Toxicol 2022; 62: 121–129. 34587458 10.1146/annurev-pharmtox-052120-020445

[26] Floris A Piga M Mangoni AA , et al. Protective effects of hydroxychloroquine against accelerated atherosclerosis in systemic lupus erythematosus. Mediators Inflamm 2018; 2018: 3424136. 29670462 10.1155/2018/3424136 PMC5835241

[27] Lorenzatti AJ Retzlaff BM . Unmet needs in the management of atherosclerotic cardiovascular disease: is there a role for emerging anti-inflammatory interventions? Int J Cardiol 2016; 221: 581–586. 27420583 10.1016/j.ijcard.2016.07.061

[28] Arnett DK Blumenthal RS Albert MA . 2019 ACC/AHA guideline on the primary prevention of cardiovascular disease: a report of the American College of Cardiology/American Heart Association Task Force on Clinical Practice Guidelines. Circulation 2019; 140: e596–e646. 10.1161/CIR.0000000000000678 PMC7734661 30879355

[29] Gubner R August S Ginsberg V . Therapeutic suppression of tissue reactivity. II. Effect of aminopterin in rheumatoid arthritis and psoriasis. Am J Med Sci 1951; 221: 176–182. 14799481

[30] Black RL O’Brien WM Vanscott EJ , et al. Methotrexate therapy in psoriatic arthritis; double-blind study on 21 patients. JAMA 1964; 189: 743–747. 14174051

[31] Vanscott EJ Auerbach R Weinstein GD . Parenteral methotrexate in psoriasis. Arch Dermatol 1964; 89: 550–556. 14107621

[32] Huennekens FM . The methotrexate story: a paradigm for development of cancer chemotherapeutic agents. Adv Enzyme Regul 1994; 34: 397–419. 7942284 10.1016/0065-2571(94)90025-6

[33] Malaviya AN . Landmark papers on the discovery of methotrexate for the treatment of rheumatoid arthritis and other systemic inflammatory rheumatic diseases: a fascinating story. Int J Rheum Dis 2016; 19: 844–851. 27293066 10.1111/1756-185X.12862

[34] Bedoui Y Guillot X Sélambarom J , et al. Methotrexate an old drug with new tricks. Int J Mol Sci 2019; 20: 20191010. 10.3390/ijms20205023 PMC6834162 31658782

[35] Cronstein BN Aune TM . Methotrexate and its mechanisms of action in inflammatory arthritis. Nat Rev Rheumatol 2020; 16: 145–154. 32066940 10.1038/s41584-020-0373-9

[36] Wilsdon TD Whittle SL Thynne TR , et al. Methotrexate for psoriatic arthritis. Cochrane Database Syst Rev 2019; 2019: CD012722. 10.1002/14651858.CD012722.pub2 PMC6353064 30656673

[37] Marks JL Edwards CJ . Protective effect of methotrexate in patients with rheumatoid arthritis and cardiovascular comorbidity. Ther Adv Musculoskelet Dis 2012; 4: 149–157. 22850632 10.1177/1759720X11436239 PMC3400102

[38] Bălănescu AR Bojincă VC Bojincă M , et al. Cardiovascular effects of methotrexate in immune-mediated inflammatory diseases. Exp Ther Med 2019; 17: 1024–1029. 30679969 10.3892/etm.2018.6992 PMC6327671

[39] van den Berg MJ Bhatt DL Kappelle LJ , et al. Identification of vascular patients at very high risk for recurrent cardiovascular events: validation of the current ACC/AHA very high risk criteria. Eur Heart J 2017; 38: 3211–3218. 28369481 10.1093/eurheartj/ehx102

[40] Silverio A Cancro FP Esposito L , et al. Secondary cardiovascular prevention after acute coronary syndrome: emerging risk factors and novel therapeutic targets. J Clin Med 2023; 12: 20230310. 10.3390/jcm12062161 PMC10056379 36983163

[41] Lechner K von Schacky C McKenzie AL , et al. Lifestyle factors and high-risk atherosclerosis: pathways and mechanisms beyond traditional risk factors. Eur J Prev Cardiol 2020; 27: 394–406. 31408370 10.1177/2047487319869400 PMC7065445

[42] Dhindsa DS Sandesara PB Shapiro MD , et al. The evolving understanding and approach to residual cardiovascular risk management. Front Cardiovasc Med 2020; 7: 88. 32478100 10.3389/fcvm.2020.00088 PMC7237700

[43] Bubb KJ Nelson AJ Nicholls SJ . Targeting triglycerides to lower residual cardiovascular risk. Expert Rev Cardiovasc Ther 2022; 20: 185–191. 35323080 10.1080/14779072.2022.2058489

[44] Ridker PM . How common is residual inflammatory risk? Circ Res 2017; 120: 617–619. 28209792 10.1161/CIRCRESAHA.116.310527

[45] Aday AW Ridker PM . Targeting residual inflammatory risk: a shifting paradigm for atherosclerotic disease. Front Cardiovasc Med 2019; 6: 16. 30873416 10.3389/fcvm.2019.00016 PMC6403155

[46] Arnold N Koenig W . Persistent inflammatory residual risk despite aggressive cholesterol-lowering therapy: what is next? Curr Opin Cardiol 2021; 36: 776–783. 34475328 10.1097/HCO.0000000000000909

[47] Liu H Wang M Xiang X , et al. Association of residual inflammatory risk with stroke recurrence in patients with acute ischaemic stroke or transient ischaemic attack. Eur J Neurol 2022; 29: 2258–2268. 35380744 10.1111/ene.15344

[48] Guedeney P Claessen BE Kalkman DN , et al. Residual inflammatory risk in patients with low LDL cholesterol levels undergoing percutaneous coronary intervention. J Am Coll Cardiol 2019; 73: 2401–2409. 31097159 10.1016/j.jacc.2019.01.077

[49] Ridker PM Bhatt DL Pradhan AD , et al. Inflammation and cholesterol as predictors of cardiovascular events among patients receiving statin therapy: a collaborative analysis of three randomised trials. J Lancet 2023; 401: 1293–1301. 10.1016/S0140-6736(23)00215-5 36893777

[50] Ridker PM Lei L Ray KK , et al. Effects of bempedoic acid on CRP, IL-6, fibrinogen and lipoprotein(a) in patients with residual inflammatory risk: a secondary analysis of the CLEAR harmony trial. J Clin Lipidol 2023; 17: 297–302. 36813656 10.1016/j.jacl.2023.02.002

[51] Adamstein NH Cornel JH Davidson M , et al. Association of interleukin 6 inhibition with ziltivekimab and the neutrophil-lymphocyte ratio: a secondary analysis of the RESCUE clinical trial. JAMA Cardiol 2023; 8: 177–181. 36449307 10.1001/jamacardio.2022.4277 PMC9713672

[52] Everett BM MacFadyen JG Thuren T , et al. Inhibition of interleukin-1β and reduction in atherothrombotic cardiovascular events in the CANTOS trial. J Am Coll Cardiol 2020; 76: 1660–1670. 33004131 10.1016/j.jacc.2020.08.011

[53] Hilvo M Wallentin L Ghukasyan Lakic T , et al. Prediction of residual risk by ceramide-phospholipid score in patients with stable coronary heart disease on optimal medical therapy. J Am Heart Assoc 2020; 9: e015258. 10.1161/JAHA.119.015258 PMC7660846 32375553

[54] Ridker PM MacFadyen JG Glynn RJ , et al. Comparison of interleukin-6, C-reactive protein, and low-density lipoprotein cholesterol as biomarkers of residual risk in contemporary practice: secondary analyses from the Cardiovascular Inflammation Reduction Trial. Eur Heart J 2020; 41: 2952–2961. 32221587 10.1093/eurheartj/ehaa160 PMC7453833

[55] Bohula EA Giugliano RP Leiter LA , et al. Inflammatory and cholesterol risk in the FOURIER trial. Circulation 2018; 138: 131–140. 29530884 10.1161/CIRCULATIONAHA.118.034032

[56] Napoli C de Nigris F Williams-Ignarro S , et al. Nitric oxide and atherosclerosis: an update. Nitric Oxide 2006; 15: 265–279. 16684613 10.1016/j.niox.2006.03.011

[57] Li H Förstermann U . Nitric oxide in the pathogenesis of vascular disease. J Pathol 2000; 190: 244–254. 10685059 10.1002/(SICI)1096-9896(200002)190:3<244::AID-PATH575>3.0.CO;2-8

[58] Cannon RO 3rd . Role of nitric oxide in cardiovascular disease: focus on the endothelium. Clin Chem 1998; 44: 1809–1819. 9702990

[59] Rudic RD Sessa WC . Nitric oxide in endothelial dysfunction and vascular remodeling: clinical correlates and experimental links. Am J Hum Genet 1999; 64: 673–677. 10052999 10.1086/302304 PMC1377782

[60] Loscalzo J . Nitric oxide insufficiency, platelet activation, and arterial thrombosis. Circ Res 2001; 88: 756–762. 11325866 10.1161/hh0801.089861

[61] Stamler JS Loh E Roddy MA , et al. Nitric oxide regulates basal systemic and pulmonary vascular resistance in healthy humans. Circulation 1994; 89: 2035–2040. 7514109 10.1161/01.cir.89.5.2035

[62] Tousoulis D Kampoli AM Tentolouris C , et al. The role of nitric oxide on endothelial function. Curr Vasc Pharmacol 2012; 10: 4–18. 22112350 10.2174/157016112798829760

[63] Wilkinson IB Franklin SS Cockcroft JR . Nitric oxide and the regulation of large artery stiffness: from physiology to pharmacology. Hypertension 2004; 44: 112–116. 15262901 10.1161/01.HYP.0000138068.03893.40

[64] Tousoulis D Oikonomou E Economou EK , et al. Inflammatory cytokines in atherosclerosis: current therapeutic approaches. Eur Heart J 2016; 37: 1723–1732. 26843277 10.1093/eurheartj/ehv759

[65] Ridker PM Rifai N Pfeffer M , et al. Elevation of tumor necrosis factor-alpha and increased risk of recurrent coronary events after myocardial infarction. Circulation 2000; 101: 2149–2153. 10801754 10.1161/01.cir.101.18.2149

[66] Yuan S Carter P Bruzelius M , et al. Effects of tumour necrosis factor on cardiovascular disease and cancer: a two-sample Mendelian randomization study. EBioMedicine 2020; 59: 102956. 32805626 10.1016/j.ebiom.2020.102956 PMC7452586

[67] Lindmark E Diderholm E Wallentin L , et al. Relationship between interleukin 6 and mortality in patients with unstable coronary artery disease: effects of an early invasive or noninvasive strategy. JAMA 2001; 286: 2107–2113. 11694151 10.1001/jama.286.17.2107

[68] Abbate A Trankle CR Buckley LF , et al. Interleukin-1 blockade inhibits the acute inflammatory response in patients with ST-segment-elevation myocardial infarction. J Am Heart Assoc 2020; 9: e014941. 10.1161/JAHA.119.014941 PMC7335541 32122219

[69] Li H Guan Y Liang B , et al. Therapeutic potential of MCC950, a specific inhibitor of NLRP3 inflammasome. Eur J Pharmacol 2022; 928: 175091. 35714692 10.1016/j.ejphar.2022.175091

[70] Qu D Guo H Xu Y . Effects of tranilast on inflammasome and macrophage phenotype in a mouse model of myocardial infarction. J Interferon Cytokine Res 2021; 41: 102–110. 33750216 10.1089/jir.2020.0208

[71] Holzer G Hoke M Sabeti-Sandor S , et al. Disparate effects of adalimumab and fumaric acid esters on cardiovascular risk factors in psoriasis patients: results from a prospective, randomized, observer-blinded head-to-head trial. J Eur Acad Dermatol Venereol 2021; 35: 441–449. 32426884 10.1111/jdv.16635

[72] Broch K Anstensrud AK Woxholt S , et al. Randomized trial of interleukin-6 receptor inhibition in patients with acute ST-segment elevation myocardial infarction. J Am Coll Cardiol 2021; 77: 1845–1855. 33858620 10.1016/j.jacc.2021.02.049

[73] Chen B Cao P Guo X , et al. Maraviroc, an inhibitor of chemokine receptor type 5, alleviates neuroinflammatory response after cerebral ischemia/reperfusion injury via regulating MAPK/NF-κB signaling. Int Immunopharmacol 2022; 108: 108755. 35395466 10.1016/j.intimp.2022.108755

[74] Gilbert J Lekstrom-Himes J Donaldson D , et al. Effect of CC chemokine receptor 2 CCR2 blockade on serum C-reactive protein in individuals at atherosclerotic risk and with a single nucleotide polymorphism of the monocyte chemoattractant protein-1 promoter region. Am J Cardiol 2011; 107: 906–911. 21247529 10.1016/j.amjcard.2010.11.005

[75] Zhao TX Kostapanos M Griffiths C , et al. Low-dose interleukin-2 in patients with stable ischaemic heart disease and acute coronary syndromes (LILACS): protocol and study rationale for a randomised, double-blind, placebo-controlled, phase I/II clinical trial. BMJ Open 2018; 8: e022452. 10.1136/bmjopen-2018-022452 PMC6144322 30224390

[76] Kim DG Lee J Seo WJ , et al. Rituximab protects against development of atherosclerotic cardiovascular disease after kidney transplantation: a propensity-matched study. Sci Rep 2019; 9: 16475–20191111. 31712593 10.1038/s41598-019-52942-8 PMC6848081

[77] Joensuu JT Aaltonen KJ Aronen P , et al. Cost-effectiveness of biologic compared with conventional synthetic disease-modifying anti-rheumatic drugs in patients with rheumatoid arthritis: a Register study. Rheumatology (Oxford, England) 2016; 55: 1803–1811. 27354689 10.1093/rheumatology/kew264

[78] Yazdany J Dudley RA Chen R , et al. Coverage for high-cost specialty drugs for rheumatoid arthritis in Medicare Part D. Arthritis Rheumatol 2015; 67: 1474–1480. 25900105 10.1002/art.39079 PMC4464809

[79] Pereira R Faria R Lago P , et al. Infection and malignancy risk in patients treated with TNF inhibitors for immune-mediated inflammatory diseases. Curr Drug Saf 2017; 12: 162–170. 28625143 10.2174/1574886312666170616085228

[80] Winthrop KL Mariette X Silva JT , et al. ESCMID Study Group for Infections in Compromised Hosts (ESGICH) Consensus Document on the safety of targeted and biological therapies: an infectious diseases perspective (soluble immune effector molecules [II]: agents targeting interleukins, immunoglobulins and complement factors). Clin Microbiol Infect 2018; 24 (Suppl. 2): S21–S40. 10.1016/j.cmi.2018.02.002 29447987

[81] Buckley LF Abbate A . Interleukin-1 blockade in cardiovascular diseases: a clinical update. Eur Heart J 2018; 39: 2063–2069. 29584915 10.1093/eurheartj/ehy128

[82] Beinsberger J Heemskerk JW Cosemans JM . Chronic arthritis and cardiovascular disease: altered blood parameters give rise to a prothrombotic propensity. Semin Arthritis Rheum 2014; 44: 345–352. 25077842 10.1016/j.semarthrit.2014.06.006

[83] Jones G Panova E . New insights and long-term safety of tocilizumab in rheumatoid arthritis. Ther Adv Musculoskelet Dis 2018; 10: 195–199. 30327685 10.1177/1759720X18798462 PMC6178374

[84] Deftereos SG Beerkens FJ Shah B , et al. Colchicine in cardiovascular disease: in-depth review. Circulation 2022; 145: 61–78. 34965168 10.1161/CIRCULATIONAHA.121.056171 PMC8726640

[85] Imazio M Nidorf M . Colchicine and the heart. Eur Heart J 2021; 42: 2745–2760. 33961006 10.1093/eurheartj/ehab221 PMC8294843

[86] Benedek TG . Methotrexate: from its introduction to non-oncologic therapeutics to anti-TNF-α. Clin Exp Rheumatol 2010; 28: S3–S8. 21044425

[87] Cipriani P Ruscitti P Carubbi F , et al. Methotrexate: an old new drug in autoimmune disease. Expert Rev Clin Immunol 2014; 10: 1519–1530. 25245537 10.1586/1744666X.2014.962996

[88] Pincus T Gibson KA Castrejón I . Update on methotrexate as the anchor drug for rheumatoid arthritis. Bull Hosp Jt Dis 2013; 71(Suppl. 1): S9–19. 24219036

[89] van Huizen AM Menting SP Gyulai R . International eDelphi study to reach consensus on the methotrexate dosing regimen in patients with psoriasis. JAMA Dermatol 2022; 158: 561–572. 35353175 10.1001/jamadermatol.2022.0434

[90] Damiani G Amerio P Bardazzi F , et al. Real-world experience of methotrexate in the treatment of skin diseases: an Italian Delphi Consensus. Dermatol Ther 2023; 13: 1219–1241. 10.1007/s13555-023-00930-2 PMC10200012 37210684

[91] Hoekstra M Haagsma C Neef C , et al. Bxotrexate comparing oral and subcutaneous administration in patients with rheumatoid arthritis. J Rheumatol 2004; 31: 645–648. 15088287

[92] Tanaka Y . Subcutaneous injection of methotrexate: advantages in the treatment of rheumatoid arthritis. Mod Rheumatol 2023; 33: 633–639. 36525530 10.1093/mr/roac156

[93] Herman RA Veng-Pedersen P Hoffman J , et al. Pharmacokinetics of low-dose methotrexate in rheumatoid arthritis patients. J Pharm Sci 1989; 78: 165–171. 2715941 10.1002/jps.2600780219

[94] Seideman P Beck O Eksborg S , et al. The pharmacokinetics of methotrexate and its 7-hydroxy metabolite in patients with rheumatoid arthritis. Br J Clin Pharmacol 1993; 35: 409–412. 8485020 10.1111/j.1365-2125.1993.tb04158.x PMC1381552

[95] Gao J Wang C Wei W . The effects of drug transporters on the efficacy of methotrexate in the treatment of rheumatoid arthritis. Life Sci 2021; 268: 118907. 33428880 10.1016/j.lfs.2020.118907

[96] Dalrymple JM Stamp LK O’Donnell JL , et al. Pharmacokinetics of oral methotrexate in patients with rheumatoid arthritis. Arthritis Rheum 2008; 58: 3299–3308. 18975321 10.1002/art.24034

[97] Inoue K Yuasa H . Molecular basis for pharmacokinetics and pharmacodynamics of methotrexate in rheumatoid arthritis therapy. Drug Metab Pharmacokinet 2014; 29: 12–19. 24284432 10.2133/dmpk.dmpk-13-rv-119

[98] Howard SC McCormick J Pui CH , et al. Preventing and managing toxicities of high-dose methotrexate. Oncologist 2016; 21: 1471–1482. 27496039 10.1634/theoncologist.2015-0164 PMC5153332

[99] Romão VC Lima A Bernardes M , et al. Three decades of low-dose methotrexate in rheumatoid arthritis: can we predict toxicity? Immunol Res 2014; 60: 289–310. 25391609 10.1007/s12026-014-8564-6

[100] Kinder AJ Hassell AB Brand J , et al. The treatment of inflammatory arthritis with methotrexate in clinical practice: treatment duration and incidence of adverse drug reactions. Rheumatology (Oxford, England) 2005; 44: 61–66. 15611303 10.1093/rheumatology/keh512

[101] Verschueren P De Cock D Corluy L , et al. Effectiveness of methotrexate with step-down glucocorticoid remission induction (COBRA Slim) versus other intensive treatment strategies for early rheumatoid arthritis in a treat-to-target approach: 1-year results of CareRA, a randomised pragmatic open-label superiority trial. Ann Rheum Dis 2017; 76: 511–520. 27432356 10.1136/annrheumdis-2016-209212

[102] Krijbolder DI Verstappen M van Dijk BT , et al. Intervention with methotrexate in patients with arthralgia at risk of rheumatoid arthritis to reduce the development of persistent arthritis and its disease burden (TREAT EARLIER): a randomised, double-blind, placebo-controlled, proof-of-concept trial. J Lancet 2022; 400: 283–294. 10.1016/S0140-6736(22)01193-X 35871815

[103] Erre GL Cadoni ML Meloni P , et al. Methotrexate therapy is not associated with increased liver stiffness and significant liver fibrosis in rheumatoid arthritis patients: a cross-sectional controlled study with real-time two-dimensional shear wave elastography. Eur J Intern Med 2019; 69: 57–63. 31474422 10.1016/j.ejim.2019.08.022

[104] Erre GL Castagna F Sauchella A , et al. Prevalence and risk factors of moderate to severe hepatic steatosis in patients with rheumatoid arthritis: an ultrasonography cross-sectional case-control study. Ther Adv Musculoskelet Dis 2021; 13: 1759720X211042739. 10.1177/1759720X211042739 PMC8606981 34819999

[105] Solomon DH Glynn RJ Karlson EW , et al. Adverse effects of low-dose methotrexate: a randomized trial. Ann Intern Med 2020; 172: 369–380. 32066146 10.7326/M19-3369 PMC7229518

[106] Wright JT Jr. Williamson JD Whelton PK , et al. A randomized trial of intensive versus standard blood-pressure control. New Engl J Med 2015; 373: 2103–2116. 26551272 10.1056/NEJMoa1511939 PMC4689591

[107] ONTARGET Investigators Yusuf S Teo KK , et al. Telmisartan, ramipril, or both in patients at high risk for vascular events. New Engl J Med 2008; 358: 1547–1559. 18378520 10.1056/NEJMoa0801317

[108] Conrad N Misra S Verbakel JY , et al. Incidence, prevalence, and co-occurrence of autoimmune disorders over time and by age, sex, and socioeconomic status: a population-based cohort study of 22 million individuals in the UK. J Lancet 2023; 401: 1878–1890. 10.1016/S0140-6736(23)00457-9 37156255

[109] Hitchon CA El-Gabalawy HS . Oxidation in rheumatoid arthritis. Arthritis Res Ther 2004; 6: 265–278. 15535839 10.1186/ar1447 PMC1064874

[110] Sparks JA . Rheumatoid arthritis. Ann Intern Med 2019; 170: ITC1–ITC16. 10.7326/AITC201901010 30596879

[111] Erre GL Paliogiannis P Castagna F , et al. Meta-analysis of neutrophil-to-lymphocyte and platelet-to-lymphocyte ratio in rheumatoid arthritis. Eur J Clin Invest 2019; 49: e13037. 10.1111/eci.13037 30316204

[112] Bassu S Zinellu A Sotgia S , et al. Oxidative stress biomarkers and peripheral endothelial dysfunction in rheumatoid arthritis: a monocentric cross-sectional case-control study. Molecules 2020; 25: 3855. 32854225 10.3390/molecules25173855 PMC7504109

[113] Erre GL Bassu S Giordo R , et al. Association between paraoxonase/arylesterase activity of serum PON-1 enzyme and rheumatoid arthritis: a systematic review and meta-analysis. Antioxidants (Basel, Switzerland) 2022; 11: 20221123. 10.3390/antiox11122317 PMC9774899 36552525

[114] Erre GL Cacciapaglia F Sakellariou G , et al. C-reactive protein and 10-year cardiovascular risk in rheumatoid arthritis. Eur J Intern Med 2022; 104: 49–54. 35821191 10.1016/j.ejim.2022.07.001

[115] Zinellu A Mangoni AA . Neutrophil-to-lymphocyte and platelet-to-lymphocyte ratio and disease activity in rheumatoid arthritis: a systematic review and meta-analysis. Eur J Clin Invest 2023; 53: e13877. 10.1111/eci.13877 36121342

[116] Bordy R Totoson P Prati C , et al. Microvascular endothelial dysfunction in rheumatoid arthritis. Nat Rev Rheumatol 2018; 14: 404–420. 29855620 10.1038/s41584-018-0022-8

[117] Mangoni AA Tommasi S Sotgia S , et al. Asymmetric dimethylarginine: a key player in the pathophysiology of endothelial dysfunction, vascular inflammation and atherosclerosis in rheumatoid arthritis? Curr Pharm Des 2021; 27: 2131–2140. 33413061 10.2174/1381612827666210106144247

[118] Erre GL Buscetta G Paliogiannis P , et al. Coronary flow reserve in systemic rheumatic diseases: a systematic review and meta-analysis. Rheumatol Int 2018; 38: 1179–1190. 29732488 10.1007/s00296-018-4039-8

[119] Erre GL Piga M Fedele AL , et al. Prevalence and determinants of peripheral microvascular endothelial dysfunction in rheumatoid arthritis patients: a multicenter cross-sectional study. Mediators Inflamm 2018; 2018: 6548715. 29483841 10.1155/2018/6548715 PMC5816852

[120] Mangoni AA Baghdadi LR Shanahan EM , et al. Methotrexate, blood pressure and markers of arterial function in patients with rheumatoid arthritis: a repeated cross-sectional study. Ther Adv Musculoskelet Dis 2017; 9: 213–229. 28932292 10.1177/1759720X17719850 PMC5600310

[121] Ambrosino P Tasso M Lupoli R , et al. Non-invasive assessment of arterial stiffness in patients with rheumatoid arthritis: a systematic review and meta-analysis of literature studies. Ann Med 2015; 47: 457–467. 26340234 10.3109/07853890.2015.1068950

[122] Arosio E De Marchi S Rigoni A , et al. Forearm haemodynamics, arterial stiffness and microcirculatory reactivity in rheumatoid arthritis. J Hypertens 2007; 25: 1273–1278. 17563541 10.1097/HJH.0b013e3280b0157e

[123] Karakulak UN Sahiner L Maharjan N , et al. Evaluation of the ambulatory arterial stiffness index in patients with rheumatoid arthritis. Blood Press Monit 2015; 20: 254–259. 25932888 10.1097/MBP.0000000000000130

[124] Woodman RJ Baghdadi LR Shanahan ME , et al. The temporal relationship between arterial stiffening and blood pressure is modified by methotrexate treatment in patients with rheumatoid arthritis. Front Physiol 2017; 8: 593. 28861004 10.3389/fphys.2017.00593 PMC5559508

[125] Jafri K Bartels CM Shin D , et al. Incidence and management of cardiovascular risk factors in psoriatic arthritis and rheumatoid arthritis: a population-based study. Arthritis Care Res 2017; 69: 51–57. 10.1002/acr.23094 PMC5191972 27696731

[126] Chen W Li S Fernandez C , et al. Temporal relationship between elevated blood pressure and arterial stiffening among middle-aged black and white adults: the Bogalusa Heart Study. Am J Epidemiol 2016; 183: 599–608. 26960706 10.1093/aje/kwv274 PMC4801137

[127] Feldmann M Brennan FM Maini RN . Role of cytokines in rheumatoid arthritis. Annu Rev Immunol 1996; 14: 397–440. 8717520 10.1146/annurev.immunol.14.1.397

[128] Tetta C Camussi G Modena V , et al. Tumour necrosis factor in serum and synovial fluid of patients with active and severe rheumatoid arthritis. Ann Rheum Dis 1990; 49: 665–667. 1700672 10.1136/ard.49.9.665 PMC1004199

[129] Kay J Calabrese L . The role of interleukin-1 in the pathogenesis of rheumatoid arthritis. Rheumatology (Oxford, England) 2004; 43(Suppl. 3): iii2–iii9. 10.1093/rheumatology/keh201 15150426

[130] Srirangan S Choy EH . The role of interleukin 6 in the pathophysiology of rheumatoid arthritis. Ther Adv Musculoskelet Dis 2010; 2: 247–256. 22870451 10.1177/1759720X10378372 PMC3383508

[131] Restivo V Candiloro S Daidone M , et al. Systematic review and meta-analysis of cardiovascular risk in rheumatological disease: symptomatic and non-symptomatic events in rheumatoid arthritis and systemic lupus erythematosus. Autoimmun Rev 2022; 21: 102925. 34454117 10.1016/j.autrev.2021.102925

[132] Baghdadi LR Woodman RJ Shanahan EM , et al. The impact of traditional cardiovascular risk factors on cardiovascular outcomes in patients with rheumatoid arthritis: a systematic review and meta-analysis. PLoS One 2015; 10: e0117952. 10.1371/journal.pone.0117952 PMC4331556 25689371

[133] Santos-Moreno P Rodríguez-Vargas GS Martínez S , et al. Metabolic abnormalities, cardiovascular disease, and metabolic syndrome in adult rheumatoid arthritis patients: current perspectives and clinical implications. Open Access Rheumatol 2022; 14: 255–267. 36388145 10.2147/OARRR.S285407 PMC9642585

[134] Xu J Xiao L Zhu J , et al. Methotrexate use reduces mortality risk in rheumatoid arthritis: a systematic review and meta-analysis of cohort studies. Semin Arthritis Rheum 2022; 55: 152031. 35671648 10.1016/j.semarthrit.2022.152031

[135] Sun KJ Liu LL Hu JH , et al. Methotrexate can prevent cardiovascular events in patients with rheumatoid arthritis: an updated meta-analysis. Medicine (Baltimore) 2021; 100: e24579. 10.1097/MD.0000000000024579 PMC7899830 33607787

[136] Baoqi Y Dan M Xingxing Z , et al. Effect of anti-rheumatic drugs on cardiovascular disease events in rheumatoid arthritis. Front Cardiovasc Med 2021; 8: 812631. 35187113 10.3389/fcvm.2021.812631 PMC8850698

[137] Rodríguez-Vargas GS Santos-Moreno P Rubio-Rubio JA , et al. Vascular age, metabolic panel, cardiovascular risk and inflammaging in patients with rheumatoid arthritis compared with patients with osteoarthritis. Front Cardiovasc Med 2022; 9: 894577. 35865390 10.3389/fcvm.2022.894577 PMC9295407

[138] Roubille C Richer V Starnino T , et al. The effects of tumour necrosis factor inhibitors, methotrexate, non-steroidal anti-inflammatory drugs and corticosteroids on cardiovascular events in rheumatoid arthritis, psoriasis and psoriatic arthritis: a systematic review and meta-analysis. Ann Rheum Dis 2015; 74: 480–489. 25561362 10.1136/annrheumdis-2014-206624 PMC4345910

[139] Xie F Chen L Yun H , et al. Benefits of methotrexate use on cardiovascular disease risk among rheumatoid arthritis patients initiating biologic disease-modifying antirheumatic drugs. J Rheumatol 2021; 48: 804–812. 33060309 10.3899/jrheum.191326

[140] Wei J Galaviz KI Kowalski AJ , et al. Comparison of cardiovascular events among users of different classes of antihypertension medications: a systematic review and network meta-analysis. JAMA Netw Open 2020; 3: e1921618. 10.1001/jamanetworkopen.2019.21618 PMC7043193 32083689

[141] Naci H Brugts JJ Fleurence R , et al. Comparative benefits of statins in the primary and secondary prevention of major coronary events and all-cause mortality: a network meta-analysis of placebo-controlled and active-comparator trials. Eur J Prev Cardiol 2013; 20: 641–657. 23447425 10.1177/2047487313480435

[142] Mangoni AA Woodman RJ Piga M , et al. Patterns of anti-inflammatory and immunomodulating drug usage and microvascular endothelial function in rheumatoid arthritis. Front Cardiovasc Med 2021; 8: 681327. 34350216 10.3389/fcvm.2021.681327 PMC8326370

[143] D’Andrea E Desai RJ He M , et al. Cardiovascular risks of hydroxychloroquine vs methotrexate in patients with rheumatoid arthritis. J Am Coll Cardiol 2022; 80: 36–46. 35772915 10.1016/j.jacc.2022.04.039 PMC9722228

[144] Ridker PM Everett BM Pradhan A , et al. Low-dose methotrexate for the prevention of atherosclerotic events. New Engl J Med 2019; 380: 752–762. 30415610 10.1056/NEJMoa1809798 PMC6587584

[145] Panja S Khatua DK Halder M . Simultaneous binding of folic acid and methotrexate to human serum albumin: insights into the structural changes of protein and the location and competitive displacement of drugs. ACS Omega 2018; 3: 246–253. 30023775 10.1021/acsomega.7b01437 PMC6045412

[146] Goldman ID Lichtenstein NS Oliverio VT . Carrier-mediated transport of the folic acid analogue, methotrexate, in the L1210 leukemia cell. J Biol Chem 1968; 243: 5007–5017. 5303004

[147] Morgan SL Baggott JE Vaughn WH , et al. Supplementation with folic acid during methotrexate therapy for rheumatoid arthritis. A double-blind, placebo-controlled trial. Ann Intern Med 1994; 121: 833–841. 7978695 10.7326/0003-4819-121-11-199412010-00002

[148] Mangoni AA Jackson SH . Homocysteine and cardiovascular disease: current evidence and future prospects. Am J Med 2002; 112: 556–565. 12015248 10.1016/s0002-9343(02)01021-5

[149] Mangoni AA Sherwood RA Asonganyi B , et al. Short-term oral folic acid supplementation enhances endothelial function in patients with type 2 diabetes. Am J Hypertens 2005; 18: 220–226. 15752950 10.1016/j.amjhyper.2004.08.036

[150] Mangoni AA Ouldred E Swif CG , et al. Vascular and blood pressure effects of folic acid in older patients with cardiovascular disease. J Am Geriatr Soc 2001; 49: 1003–1004. 11527499 10.1046/j.1532-5415.2001.49196.x

[151] Mangoni AA Sherwood RA Swift CG , et al. Folic acid enhances endothelial function and reduces blood pressure in smokers: a randomized controlled trial. J Intern Med 2002; 252: 497–503. 12472909 10.1046/j.1365-2796.2002.01059.x

[152] Huo Y Li J Qin X , et al. Efficacy of folic acid therapy in primary prevention of stroke among adults with hypertension in China: the CSPPT randomized clinical trial. JAMA 2015; 313: 1325–1335. 25771069 10.1001/jama.2015.2274

[153] Gomes FLT Maranhão RC Tavares ER , et al. Regression of atherosclerotic plaques of cholesterol-fed rabbits by combined chemotherapy with paclitaxel and methotrexate carried in lipid core nanoparticles. J Cardiovasc Pharmacol Ther 2018; 23: 561–569. 29779420 10.1177/1074248418778836

[154] Mašić D Stengaard-Pedersen K Løgstrup BB , et al. Similar lipid level changes in early rheumatoid arthritis patients following 1-year treat-to-target strategy with adalimumab plus methotrexate versus placebo plus methotrexate: secondary analyses from the randomised controlled OPERA trial. Rheumatol Int 2021; 41: 543–549. 33386898 10.1007/s00296-020-04756-5

[155] Enrich C Rentero C de Muga SV , et al. Annexin A6-linking Ca(2+) signaling with cholesterol transport. Biochim Biophys Acta 2011; 1813: 935–947. 20888375 10.1016/j.bbamcr.2010.09.015

[156] Zhang L Spencer KL Voruganti VS , et al. Association of functional polymorphism rs2231142 (Q141K) in the ABCG2 gene with serum uric acid and gout in 4 US populations: the PAGE Study. Am J Epidemiol 2013; 177: 923–932. 23552988 10.1093/aje/kws330 PMC4023295

[157] Wang B Deng H Hu Y , et al. The difference of lipid profiles between psoriasis with arthritis and psoriasis without arthritis and sex-specific downregulation of methotrexate on the apolipoprotein B/apolipoprotein A-1 ratio. Arthritis Res Ther 2022; 24: 17. 34996506 10.1186/s13075-021-02715-4 PMC8740478

[158] Krahel JA Baran A Kamiński TW , et al. Methotrexate decreases the level of PCSK9-A novel indicator of the risk of proatherogenic lipid profile in psoriasis. The preliminary data. J Clin Med 2020; 9: 20200326. 10.3390/jcm9040910 PMC7230388 32225075

[159] Artenstein AW Opal SM . Proprotein convertases in health and disease. New Engl J Med 2011; 365: 2507–2518. 22204726 10.1056/NEJMra1106700

[160] Coppinger C Movahed MR Azemawah V , et al. A comprehensive review of PCSK9 inhibitors. J Cardiovasc Pharmacol Ther 2022; 27: 10742484221100107. 35593194 10.1177/10742484221100107

[161] Baghdadi LR . Effect of methotrexate use on the development of type 2 diabetes in rheumatoid arthritis patients: a systematic review and meta-analysis. PLoS One 2020; 15: e0235637. 10.1371/journal.pone.0235637 PMC7337336 32628710

[162] Xie W Yang X Ji L , et al. Incident diabetes associated with hydroxychloroquine, methotrexate, biologics and glucocorticoids in rheumatoid arthritis: a systematic review and meta-analysis. Semin Arthritis Rheum 2020; 50: 598–607. 32480098 10.1016/j.semarthrit.2020.04.005

[163] Nam SH Kim M Kim YJ , et al. Risk of new-onset diabetes mellitus associated with antirheumatic drugs in patients with rheumatoid arthritis: a nationwide population study. J Clin Med 2022; 11: 20220410. 10.3390/jcm11082109 PMC9026381 35456202

[164] Baker JF Sauer B Teng CC , et al. Initiation of disease-modifying therapies in rheumatoid arthritis is associated with changes in blood pressure. J Clin Rheumatol 2018; 24: 203–209. 29664818 10.1097/RHU.0000000000000736 PMC7461421

[165] Toms TE Panoulas VF John H , et al. Methotrexate therapy associates with reduced prevalence of the metabolic syndrome in rheumatoid arthritis patients over the age of 60- more than just an anti-inflammatory effect? A cross sectional study. Arthritis Res Ther 2009; 11: R110. 10.1186/ar2765 PMC2745792 19607680

[166] Bilecik NA Tuna S Samancı N , et al. Prevalence of metabolic syndrome in women with rheumatoid arthritis and effective factors. Int J Clin Exp Med 2014; 7: 2258–2265. 25232418 PMC4161578

[167] Costa L Caso F Atteno M , et al. Impact of 24-month treatment with etanercept, adalimumab, or methotrexate on metabolic syndrome components in a cohort of 210 psoriatic arthritis patients. Clin Rheumatol 2014; 33: 833–839. 23959447 10.1007/s10067-013-2369-1

[168] Seitz M Zwicker M Loetscher P . Effects of methotrexate on differentiation of monocytes and production of cytokine inhibitors by monocytes. Arthritis Rheum 1998; 41: 2032–2038. 9811059 10.1002/1529-0131(199811)41:11<2032::AID-ART19>3.0.CO;2-J

[169] Edwards CK 3rd Bendele AM Reznikov LI , et al. Soluble human p55 and p75 tumor necrosis factor receptors reverse spontaneous arthritis in transgenic mice expressing transmembrane tumor necrosis factor alpha. Arthritis Rheum 2006; 54: 2872–2885. 16947419 10.1002/art.22077

[170] Yamasaki E Soma Y Kawa Y , et al. Methotrexate inhibits proliferation and regulation of the expression of intercellular adhesion molecule-1 and vascular cell adhesion molecule-1 by cultured human umbilical vein endothelial cells. Br J Dermatol 2003; 149: 30–38. 10.1046/j.1365-2133.2003.05407.x 12890192

[171] Quan A Pan Y Singh KK , et al. Cardiovascular inflammation is reduced with methotrexate in diabetes. Mol Cell Biochem 2017; 432: 159–167. 28303409 10.1007/s11010-017-3006-0

[172] Ma Y Li L Shao Y , et al. Methotrexate improves perivascular adipose tissue/endothelial dysfunction via activation of AMPK/eNOS pathway. Mol Med Rep 2017; 15: 2353–2359. 28259947 10.3892/mmr.2017.6225

[173] Municio C Dominguez-Soto Á Fuentelsaz-Romero S , et al. Methotrexate limits inflammation through an A20-dependent cross-tolerance mechanism. Ann Rheum Dis 2018; 77: 752–759. 29431121 10.1136/annrheumdis-2017-212537 PMC5909749

[174] Stigliano C Ramirez MR Singh JV , et al. Methotrexate-loaded hybrid nanoconstructs target vascular lesions and inhibit atherosclerosis progression in ApoE(-/-) mice. Adv Healthcare Mater 2017; 6: 1601286. 10.1002/adhm.201601286 28402587

[175] Mangoni AA Zinellu A Sotgia S , et al. Protective effects of methotrexate against proatherosclerotic cytokines: a review of the evidence. Mediators Inflamm 2017; 2017: 9632846. 29430085 10.1155/2017/9632846 PMC5753000

[176] Owczarczyk-Saczonek A Drozdowski M Maciejewska-Radomska A , et al. The effect of subcutaneous methotrexate on markers of metabolic syndrome in psoriatic patients – preliminary report. Postepy Dermatol Alergol 2018; 35: 53–59. 29599672 10.5114/ada.2017.71358 PMC5872240

[177] Gong K Zhang Z Sun X , et al. The nonspecific anti-inflammatory therapy with methotrexate for patients with chronic heart failure. Am Heart J 2006; 151: 62–68. 16368293 10.1016/j.ahj.2005.02.040

[178] Zhang R Chen S Zhang H , et al. Effects of methotrexate in a rabbit model of in-stent neoatherosclerosis: an optical coherence tomography study. Sci Rep 2016; 6: 33657. 27644847 10.1038/srep33657 PMC5028880

[179] Fiorelli AI Lourenço-Filho DD Tavares ER , et al. Methotrexate associated to lipid core nanoparticles improves cardiac allograft vasculopathy and the inflammatory profile in a rabbit heart graft model. Braz J Med Biol Res 2017; 50: e6225. 10.1590/1414-431X20176225 PMC5561808 28832763

[180] Zhang Z Zhao P Li A , et al. Effects of methotrexate on plasma cytokines and cardiac remodeling and function in postmyocarditis rats. Mediators Inflamm 2009; 2009: 389720. 19884981 10.1155/2009/389720 PMC2768010

[181] DeOliveira CC Acedo SC Gotardo EM . Effects of methotrexate on inflammatory alterations induced by obesity: an in vivo and in vitro study. Mol Cell Endocrinol 2012; 361: 92–98. 22480543 10.1016/j.mce.2012.03.016

[182] Saleem MU Muhammad F Sharif A , et al. Methotrexate-loaded biodegradable nanoparticles exert anti-arthritic effect by downregulating pro-inflammatory cytokines in Freund’s complete adjuvant-induced arthritic rats. Inflammopharmacology 2022; 30: 1079–1091. 35426539 10.1007/s10787-022-00977-1

[183] Stähli A Scherler C Zappalà G , et al. In vitro activity of anti-rheumatic drugs on release of pro-inflammatory cytokines from oral cells in interaction with microorganisms. Front Oral Health 2022; 3: 960732. 36118051 10.3389/froh.2022.960732 PMC9478466

[184] Zdanowska N Owczarczyk-Saczonek A Czerwińska J , et al. Adalimumab and methotrexate affect the concentrations of regulatory cytokines (interleukin-10, transforming growth factor-β1, and interleukin-35) in patients with plaque psoriasis. Dermatol Ther 2020; 33: e14153. 10.1111/dth.14153 32770629

[185] Ridker PM Everett BM Thuren T , et al. Antiinflammatory therapy with canakinumab for atherosclerotic disease. New Engl J Med 2017; 377: 1119–1131. 28845751 10.1056/NEJMoa1707914

[186] Iwamoto T Umemura S Toya Y , et al. Identification of adenosine A2 receptor-camp system in human aortic endothelial cells. Biochem Biophys Res Commun 1994; 199: 905–910. 8135838 10.1006/bbrc.1994.1314

[187] Ansari HR Nadeem A Talukder MA , et al. Evidence for the involvement of nitric oxide in A2B receptor-mediated vasorelaxation of mouse aorta. Am J Physiol Heart Circ Physiol 2007; 292: H719–H725. 10.1152/ajpheart.00593.2006 16920807

[188] Tang L Parker M Fei Q , et al. Afferent arteriolar adenosine A2a receptors are coupled to KATP in in vitro perfused hydronephrotic rat kidney. Am J Physiol 1999; 277: F926–F933. 10.1152/ajprenal.1999.277.6.F926 10600940

[189] Maimon N Titus PA Sarelius IH . Pre-exposure to adenosine, acting via A(2A) receptors on endothelial cells, alters the protein kinase A dependence of adenosine-induced dilation in skeletal muscle resistance arterioles. J Physiol 2014; 592: 2575–2590. 24687580 10.1113/jphysiol.2013.265835 PMC4080939

[190] Zou AP Nithipatikom K Li PL , et al. Role of renal medullary adenosine in the control of blood flow and sodium excretion. Am J Physiol 1999; 276: R790–R798. 10.1152/ajpregu.1999.276.3.R790 10070140

[191] Schindler CW Karcz-Kubicha M Thorndike EB , et al. Role of central and peripheral adenosine receptors in the cardiovascular responses to intraperitoneal injections of adenosine A1 and A2A subtype receptor agonists. Br J Pharmacol 2005; 144: 642–650. 15678095 10.1038/sj.bjp.0706043 PMC1576042

[192] Mahmud A Feely J . Acute effect of caffeine on arterial stiffness and aortic pressure waveform. Hypertension 2001; 38: 227–231. 11509481 10.1161/01.hyp.38.2.227

[193] Koupenova M Johnston-Cox H Ravid K . Regulation of atherosclerosis and associated risk factors by adenosine and adenosine receptors. Curr Atheroscler Rep 2012; 14: 460–468. 22850979 10.1007/s11883-012-0263-y PMC3759396

[194] Varani K Portaluppi F Merighi S , et al. Caffeine alters A2A adenosine receptors and their function in human platelets. Circulation 1999; 99: 2499–2502. 10330379 10.1161/01.cir.99.19.2499

[195] Haskó G Pacher P . Regulation of macrophage function by adenosine. Arterioscler Thromb Vasc Biol 2012; 32: 865–869. 22423038 10.1161/ATVBAHA.111.226852 PMC3387535

[196] Voloshyna I Reiss AB . The ABC transporters in lipid flux and atherosclerosis. Prog Lipid Res 2011; 50: 213–224. 21352852 10.1016/j.plipres.2011.02.001

[197] Escher G Krozowski Z Croft KD , et al. Expression of sterol 27-hydroxylase (CYP27A1) enhances cholesterol efflux. J Biol Chem 2003; 278: 11015–11019. 12531903 10.1074/jbc.M212780200

[198] Li J Meng Q Fu Y , et al. Novel insights: dynamic foam cells derived from the macrophage in atherosclerosis. J Cell Physiol 2021; 236: 6154–6167. 33507545 10.1002/jcp.30300

[199] Višnjić D Lalić H Dembitz V , et al. AICAr, a widely used AMPK activator with important AMPK-independent effects: a systematic review. Cells 2021; 10: 1095. 34064363 10.3390/cells10051095 PMC8147799

[200] Hardie DG . AMP-activated protein kinase: an energy sensor that regulates all aspects of cell function. Genes Dev 2011; 25: 1895–1908. 21937710 10.1101/gad.17420111 PMC3185962

[201] Antonioli L Colucci R Pellegrini C , et al. The AMPK enzyme-complex: from the regulation of cellular energy homeostasis to a possible new molecular target in the management of chronic inflammatory disorders. Expert Opin Ther Targets 2016; 20: 179–191. 26414111 10.1517/14728222.2016.1086752

[202] Boin F Erre GL Posadino AM , et al. Oxidative stress-dependent activation of collagen synthesis is induced in human pulmonary smooth muscle cells by sera from patients with scleroderma-associated pulmonary hypertension. Orphanet J Rare Dis 2014; 9: 123. 25085432 10.1186/s13023-014-0123-7 PMC4237898

[203] Igata M Motoshima H Tsuruzoe K , et al. Adenosine monophosphate-activated protein kinase suppresses vascular smooth muscle cell proliferation through the inhibition of cell cycle progression. Circ Res 2005; 97: 837–844. 16151020 10.1161/01.RES.0000185823.73556.06

[204] Li J Wang Y Wang Y , et al. Pharmacological activation of AMPK prevents drp1-mediated mitochondrial fission and alleviates endoplasmic reticulum stress-associated endothelial dysfunction. J Mol Cell Cardiol 2015; 86: 62–74. 26196303 10.1016/j.yjmcc.2015.07.010

[205] Chen Z Peng IC Sun W , et al. AMP-activated protein kinase functionally phosphorylates endothelial nitric oxide synthase Ser633. Circ Res 2009; 104: 496–505. 19131647 10.1161/CIRCRESAHA.108.187567 PMC2761102

[206] Goirand F Solar M Athea Y , et al. Activation of AMP kinase alpha1 subunit induces aortic vasorelaxation in mice. J Physiol 2007; 581: 1163–1171. 17446219 10.1113/jphysiol.2007.132589 PMC2170850

[207] Nagata D Takeda R Sata M , et al. AMP-activated protein kinase inhibits angiotensin II-stimulated vascular smooth muscle cell proliferation. Circulation 2004; 110: 444–451. 15262850 10.1161/01.CIR.0000136025.96811.76

[208] Ford RJ Teschke SR Reid EB , et al. AMP-activated protein kinase activator AICAR acutely lowers blood pressure and relaxes isolated resistance arteries of hypertensive rats. J Hypertens 2012; 30: 725–733. 22306847 10.1097/HJH.0b013e32835050ca

[209] Buhl ES Jessen N Pold R , et al. Long-term AICAR administration reduces metabolic disturbances and lowers blood pressure in rats displaying features of the insulin resistance syndrome. Diabetes 2002; 51: 2199–2206. 12086950 10.2337/diabetes.51.7.2199

[210] Samsonov MV Podkuychenko NV Khapchaev AY , et al. AICAR protects vascular endothelial cells from oxidative injury induced by the long-term palmitate excess. Int J Mol Sci 2021; 23: 211. 35008640 10.3390/ijms23010211 PMC8745318

[211] Pang ZD Wang Y Song Z , et al. AMPK upregulates K(Ca)2.3 channels and ameliorates endothelial dysfunction in diet-induced obese mice. Biochem Pharmacol 2021; 183: 114337. 33186592 10.1016/j.bcp.2020.114337

[212] Angin Y Beauloye C Horman S , et al. Regulation of carbohydrate metabolism, lipid metabolism, and protein metabolism by AMPK. Exp Suppl 2016; 107: 23–43. 27812975 10.1007/978-3-319-43589-3_2

[213] Steinberg GR Hardie DG . New insights into activation and function of the AMPK. Nat Rev Mol Cell Biol 2023; 24: 255–272. 36316383 10.1038/s41580-022-00547-x

[214] Heidary Moghaddam R Samimi Z Asgary S , et al. Natural AMPK activators in cardiovascular disease prevention. Front Pharmacol 2021; 12: 738420. 35046800 10.3389/fphar.2021.738420 PMC8762275

[215] Cronstein BN Naime D Ostad E . The antiinflammatory mechanism of methotrexate. Increased adenosine release at inflamed sites diminishes leukocyte accumulation in an in vivo model of inflammation. J Clin Investig 1993; 92: 2675–2682. 8254024 10.1172/JCI116884 PMC288465

[216] Hess JA Khasawneh MK . Cancer metabolism and oxidative stress: insights into carcinogenesis and chemotherapy via the non-dihydrofolate reductase effects of methotrexate. BBA Clin 2015; 3: 152–161. 26674389 10.1016/j.bbacli.2015.01.006 PMC4661551

[217] Duryee MJ Klassen LW Schaffert CS , et al. Malondialdehyde-acetaldehyde adduct is the dominant epitope after MDA modification of proteins in atherosclerosis. Free Radic Biol Med 2010; 49: 1480–1486. 20696236 10.1016/j.freeradbiomed.2010.08.001 PMC2952714

[218] Zimmerman MC Clemens DL Duryee MJ , et al. Direct antioxidant properties of methotrexate: inhibition of malondialdehyde-acetaldehyde-protein adduct formation and superoxide scavenging. Redox Biol 2017; 13: 588–593. 28803127 10.1016/j.redox.2017.07.018 PMC5552384

[219] Luna-López A Flores-González GA Rivera-Ruz IA , et al. Methotrexate induces an antioxidant hormetic response in primary rat astrocytes. Dose Response 2022; 20: 15593258221130752. 36325182 10.1177/15593258221130752 PMC9619289

[220] Chowdhury R Khan H Heydon E , et al. Adherence to cardiovascular therapy: a meta-analysis of prevalence and clinical consequences. Eur Heart J 2013; 34: 2940–2948. 23907142 10.1093/eurheartj/eht295

[221] Jekic B Maksimovic N Damnjanovic T . Methotrexate pharmacogenetics in the treatment of rheumatoid arthritis. Pharmacogenomics 2019; 20: 1235–1245. 31648623 10.2217/pgs-2019-0121

[222] Campbell JM Bateman E Stephenson MD , et al. Methotrexate-induced toxicity pharmacogenetics: an umbrella review of systematic reviews and meta-analyses. Cancer Chemother Pharmacol 2016; 78: 27–39. 27142726 10.1007/s00280-016-3043-5

[223] Szostak B Machaj F Rosik J , et al. Using pharmacogenetics to predict methotrexate response in rheumatoid arthritis patients. Expert Opin Drug Metab Toxicol 2020; 16: 617–626. 32500745 10.1080/17425255.2020.1777279

[224] Baghdadi LR Woodman RJ Shanahan EM , et al. Genetic polymorphism of the methotrexate transporter ABCG2, blood pressure and markers of arterial function in patients with rheumatoid arthritis: repeated cross-sectional study. Pharmgenomics Pers Med 2018; 11: 205–210. 30519074 10.2147/PGPM.S170557 PMC6237132

[225] Nidorf SM Eikelboom JW Budgeon CA , et al. Low-dose colchicine for secondary prevention of cardiovascular disease. J Am Coll Cardiol 2013; 61: 404–410. 23265346 10.1016/j.jacc.2012.10.027

[226] Sharma TS Wasko MC Tang X , et al. Hydroxychloroquine use is associated with decreased incident cardiovascular events in rheumatoid arthritis patients. J Am Heart Assoc 2016; 5: e002867. 10.1161/JAHA.115.002867 PMC4859400 26727968

[227] Shapiro M Levy Y . The association between hydroxychloroquine treatment and cardiovascular morbidity among rheumatoid arthritis patients. Oncotarget 2018; 9: 6615–6622. 29464097 10.18632/oncotarget.23570 PMC5814237

